# Efficient Synthesis of Novel Propargyl Sulfamate Derivatives and Their Potent Inhibitory Effects on Human Carbonic Anhydrase Isoenzymes

**DOI:** 10.1021/acsomega.6c02129

**Published:** 2026-06-18

**Authors:** Ufuk Atmaca, Songül Bayrak, Cetin Bayrak

**Affiliations:** 1 Department of Chemistry, Faculty of Science, Atatürk University, Erzurum 25240, Turkey; 2 Doǧubayazıt Ahmed-i Hani Vocational School, Aǧrı İbrahim Çeçen University, Agri 04400, Turkey

## Abstract

In the present study, a total of 21 novel propargyl sulfamate derivatives were designed and synthesized as potential inhibitors of human carbonic anhydrases I and II (hCA I and hCA II). An efficient and practical synthetic protocol was developed based on the reaction of propargyl alcohols with chlorosulfonyl isocyanate (CSI), affording the target sulfamate compounds in good yield. The inhibitory activities of all synthesized compounds against hCA I and hCA II isoenzymes were evaluated in vitro. The compounds exhibited potent inhibition with IC_50_ values ranging from 8.97 to 161.16 nM for hCA I and from 3.89 to 177.69 nM for hCA II. Among the tested molecules, methyl (1-(2,5-dimethoxyphenyl)-3-phenylprop-2-yn-1-yl)­sulfamate (**2i**) emerged as the most active derivative, displaying superior inhibitory activity compared to the reference drug acetazolamide (AZA), with IC_50_ values of 8.97 and 3.89 nM against hCA I and hCA II, respectively. Compound **2i** is 7.72 times more active than the standard drug AZA in inhibiting the hCA I isoform and 15.35 times more active in inhibiting the hCA II isoform. Furthermore, selected compounds (**2i**, **2h**, and **2b**) were evaluated against tumor-associated isoenzymes hCA IX and hCA XII, exhibiting strong inhibition with IC_50_ values ranging from 5.12 to 26.85 nM. Notably, compound **2i** demonstrated the highest activity with IC_50_ values of 6.45 nM for hCA IX and 5.12 nM for hCA XII, surpassing the reference inhibitor AZA. These findings indicated that **2i**, because of its strong carbonic anhydrase inhibition effect, represented a promising candidate for further drug development. This methodology may also serve as a convenient strategy for the generation of cinnamaldehyde derivatives.

## Graphical abstract

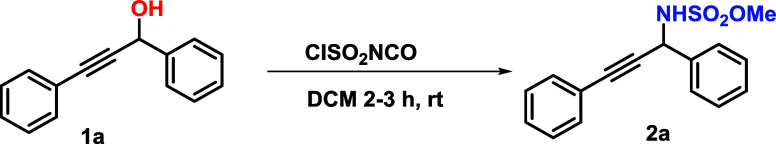

## 1 Introduction

Carbonic anhydrases (CAs; carbonate hydrolyzers; EC 4.2.1.1) are zinc-containing metalloenzymes that reversibly catalyze the hydration of carbon dioxide to bicarbonate ions and protons in the human body.^1,2^ To date, eight independent carbonic anhydrase gene families have been identified; however, only α-carbonic anhydrase (α-CA) isoforms are present in humans. Thus far, 15 distinct human α-CA isoenzymes have been characterized.^3,4^ All human α-CAs possess a conserved active site in which a Zn^+2^ ion is coordinated by three histidine residues and a water molecule or hydroxide ion.^5,6^ Previous studies have demonstrated that these isoforms differ considerably in their molecular properties, tissue and organ distributions, cellular localizations, expression patterns, and sensitivities to various classes of inhibitors.^7,8^ Carbonic anhydrases catalyze a wide range of reactions in humans,^9,10^ and these CA-mediated processes play essential roles in numerous physiological and pathological conditions.^11,12^ In recent years, intensified research on carbonic anhydrases and the identification of selective inhibitors have significantly contributed to the treatment of several diseases, including glaucoma, cancer, obesity, and epilepsy. Consequently, carbonic anhydrase inhibitors (CAIs) are regarded as important therapeutic agents. A sulfamate moiety is a privileged group in the medicine, drug chemistry, and material sciences. They are found in the structure of many medicines and have recently emerged as valuable anticancer agents.^13–15^ They are also mostly associated with carbonic anhydrases, which are vital for human health, and have promising inhibitory effects against most CA isoforms.^16–18^ Amines are used in chemistry as intermediates in the production of many polymers and dyes. They are found in many natural product structures, such as amino acids, alkaloids, and nucleotides. The N-substituted amine group is found in many important drugs, such as fluoxetine, duloxetine, desipramine, and epinephrine.^19,20^ Synthesis of amines or protected amines from alcohols is one of the most widely known methods in the literature. In these syntheses, amine production involves challenging steps, such as alcohol activation and nucleophilic displacement.^21^ The conversion of alcohol to amine has long been known (see Scheme 1a). The synthesis of propargyl amine and its derivatives was reported by Sakai et al. The propargyl amine and its derivatives can be formed from the reaction of a protected N-benzylic compound with acetylene derivatives in the presence of InBr_3_/Et_3_N (Scheme 1b).^22^ Samai et al. reported that they synthesized propargylamine from aldehydes, amines, and alkynes via a three-component coupling reaction with a NiCl_2_ catalyst (Scheme 1c).^23^ In our study, we disclosed that novel propargyl sulfamates can be synthesized from propargyl alcohols in a single process without using any catalyst, effective on a wide range of substrates, including different functional groups (Scheme 1d). The inhibitory activities of all synthesized compounds against hCA I and hCA II isoenzymes were evaluated in vitro.

**Scheme 1:**
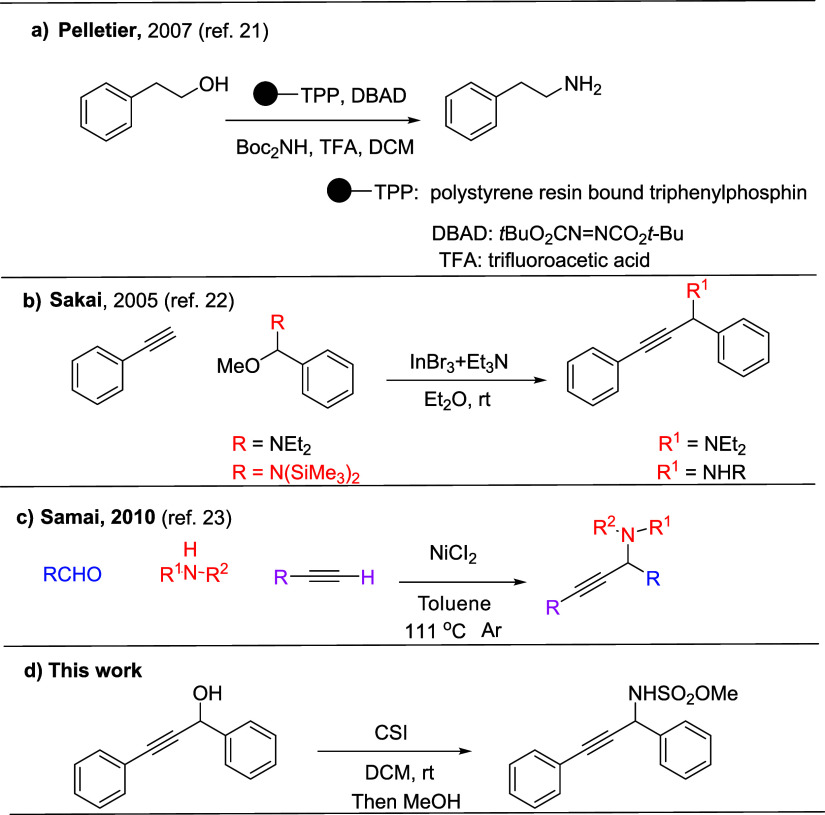
Synthesis of Amine and Propargyl Amine Derivatives: (a) Pelletier, 2007 (Reference 21), (b) Sakai, 2005 (Reference 22), (c) Samai, 2010 (Reference 23), and (d) This Work

## 2 Result and Discussion

### 2.1 Chemistry

To start our investigations, we focused on reactions between propargyl alcohols and CSl. We have synthesized propargyl alcohols using a known method.^24^ We began our studies with propargyl alcohols (**1a**) and CSl as the model substrates to screen the reaction conditions, and after optimization, our best reaction conditions are defined in Table 1. Initially, we began the reaction with propargylic alcohols (**1a**) and CSl in the presence of lewis acids, such as BF_3_, AlCI_3_, and LiCIO_4_, with different additives, such as H_2_O, EtOH, and MeOH (Table 1, entries 1–5); no product could be detected. The reaction was carried out without the presence of a Lewis acid and EtOH in place of MeOH (Table 1, entry 6); however, the desired product did not occurred. MeOH is an important additive for the reaction. Thus, it should be used in the reaction. The desired product **2a** was isolated in 49% yield in the presence of trifluoroacetic acid (TFA) with an additive of MeOH (Table 1, entry 7). Evaluation of the solvents smoothly promoted the reaction efficiency, delivering **2a** in 61–85% yields in Scheme 2 (Table 1, entries 8 and 9).

**Scheme 2:**
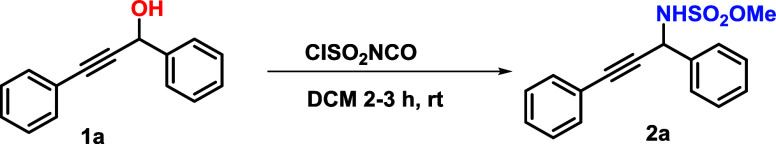
Standard Reaction of Propargyl Alcohol with CSI

**Table 1:** Optimization of Reaction Conditions

entry	substrate	solvent	additive	time (h)	yield (%)
1	**1a**	CH_2_CI_2_	BF_3_/H_2_O	2	0
2	**1a**	CH_2_CI_2_	BF_3_/MeOH	2	0
3	**1a**	CH_2_CI_2_	AlCI_3_/H_2_O	2	0
4	**1a**	CH_2_CI_2_	AlCI_3_/MeOH	2	0
5	**1a**	CH_2_CI_2_	LiCIO_4_/MeOH	2	0
6	**1a**	CH_2_CI_2_	EtOH	2	0
7	**1a**	CH_2_CI_2_	TFA/MeOH	2	49
8	**1a**	CH_3_CN	MeOH	2	61
**9**	**1a**	**CH**_**2**_ **CI**_**2**_	**MeOH**	**2**	**85**

With the best optimization conditions (Table 1, entry 9) in hand, the scope of phenylacetylene substrates was reviewed and is summarized in Table 1. Aromatic propargyl alcohols with different substituent groups were studied and efficiently converted to the corresponding propargyl sulfamate. The aromatic substrate without substituents (**1a**) and electron-rich aromatic substrates containing methyl at different positions (**1b**–**d**) were converted to the corresponding propargyl sulfamate in good yields (72–83%). Similarly, substrates (**1h** and **1i**) containing one or two OMe groups gave the corresponding compound in the good yields (82 and 85% respectively). Electron-deficient aromatic substrates (**1e**–**g**) containing halogens (F, Cl, and Br) at the same position also gave the corresponding propargyl sulfamate in excellent yields (79–84%). However, the aromatic substrate containing an electron-withdrawing NO_2_ group (**2j**) gave a decreased yield (58%). Aromatic substrates containing groups such as thiophene and naphthalene were efficiently converted to the corresponding propargyl sulfamate in Scheme 3 (68 and 72%, respectively).

**Scheme 3:**
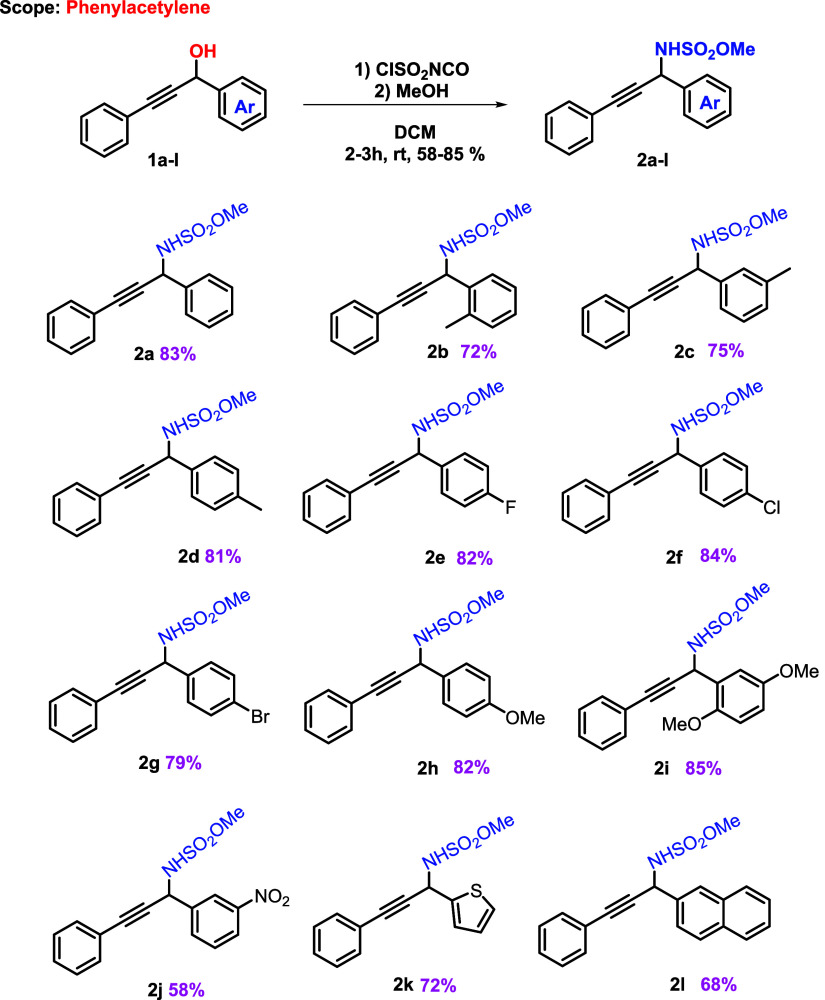
Synthesis of Novel Propargyl Sulfamates (**2a**–**2l**)

The scope was extended to *p*-methyl-substituted propargyl alcohols (**3a**–**g**) with CSI. Phenyl-, *o*-methylphenyl-, and *m*-methylphenyl-substituted phenyl propargyl alcohols were reacted separately with CSI, and the desired products (**4a**–**c**) were obtained in excellent yields (85, 75, and 78%, respectively). Additionally, chlorine, fluorine, and bromine at the *para* positions of substituted phenyl propargyl alcohols (**4d**–**f**) also gave the desired products with good yields in Scheme 4 (83, 78, and 82%, respectively).

**Scheme 4:**
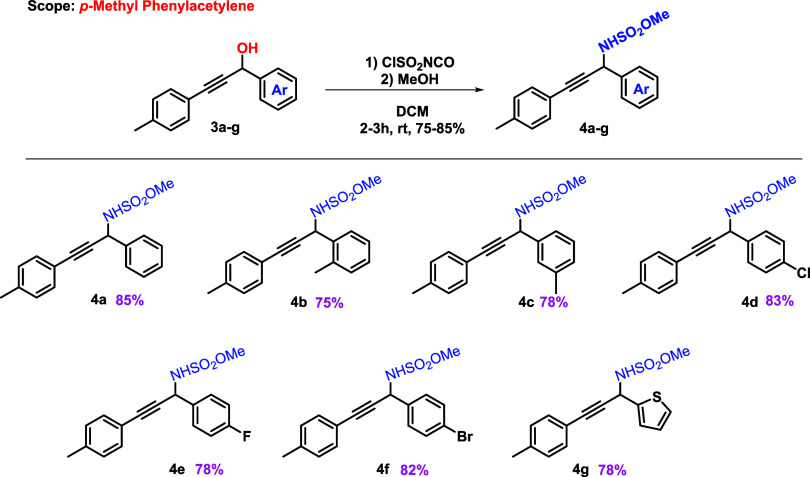
Synthesis of Novel Propargyl Sulfamates (**4a**–**4g**)

To verify larger-scale synthesis of methyl (1,3-diphenylprop-2-yn-1-yl)­sulfamate (**2a**). The scaled-up experiment was carried out using propargyl alcohol (**1a**) (1 g, 4.8 mmol) and successfully obtained **2a** (1.13 g, 78% yield) with an insignificant decrease in yield (Scheme 5).

**Scheme 5:**
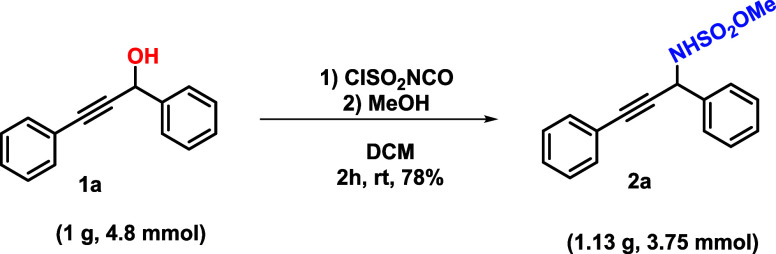
Larger-Scale Synthesis of **2a**

Cinnamaldehyde serves as the key active constituent of the spice cinnamon. It is important due to its anti-inflammatory, antimicrobial, antioxidant, antitumor, hypocholesterolemic, and immunomodulatory effects.^25^ That is why cinnamaldehyde derivative (**1m**) was reacted with CSI, and two isomers were isolated. The first isomer (**2m**) is a standard adduct product, and the second isomer (**2n**) is a rearrangement product (Scheme 6). This method may serve as a convenient strategy for generating cinnamaldehyde derivatives.

**Scheme 6:**
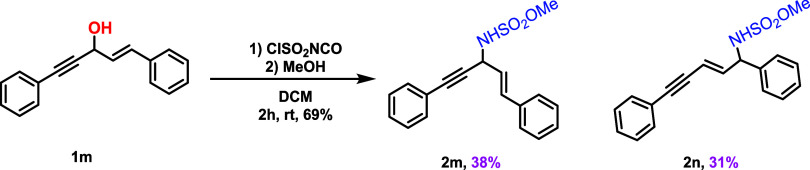
Application for the Cinnamaldehyde Derivative

A plausible mechanism for the formation of propargyl sulfamates from propargyl alcohols and CSI is proposed and depicted in Scheme 7. In the presence of MeOH, acting as a hydrogen-bond donor, activation of the propargyl alcohol **1a** leads to cleavage of the C–OH bond, generating a stabilized propargylic cation intermediate. This cation subsequently undergoes a nucleophilic attack by CSI, resulting in C–N bond formation to afford intermediate **II**. Next, intermediate **II** undergoes CO_2_ elimination, producing the sulfonyl-stabilized cationic species **III**. Finally, proton transfer and S–OMe bond formation occur, leading to rearrangement of intermediate **III** and furnishing the final propargyl sulfamate product **2a**.

**Scheme 7:**
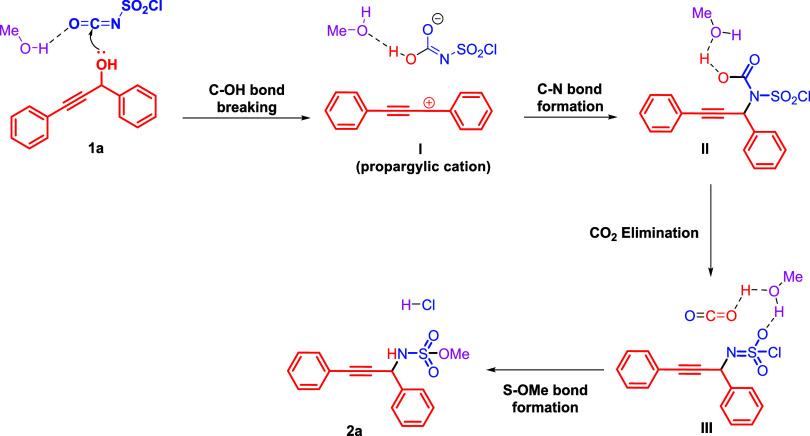
Possible Reaction Mechanism

### 2.2 Inhibition Experiment of Carbonic Anhydrase Enzymes

Carbonic anhydrase enzymes are important metalloenzymes that catalyze the reversible hydration of CO_2_ and play a key role in acid–base balance and ion transport. In this study, the inhibitory effects of 22 newly synthesized sulfamate derivatives on hCA I and hCA II were evaluated (Table 2 and Scheme 8). All compounds inhibited both isoenzymes at the nanomolar level, with IC_50_ values ranging from 3.89 to 177.69 nM. To further assess isoform selectivity, the most active compounds (**2i**, **2h**, and **2b**) were additionally evaluated against tumor-associated carbonic anhydrase isoforms hCA IX and hCA XII (Table 3). The IC_50_ values for these isoforms ranged from 5.12 to 26.85 nM, indicating that the selected compounds also exhibit strong inhibition of cancer-related isoforms, with compound **2i** showing superior activity compared to the reference inhibitor, acetazolamide.

**Table 2:** Inhibitory Effects and IC_50_ Values of Synthesized Sulfamate Derivatives against Human Carbonic Anhydrase I and II^a^

	IC_50_ (nM)			
compounds	hCA I	*R* ^2^	hCA II	***R*** ^2^
**2a**	45.89	0.992	39.60	0.993
**2b**	30.13	0.996	19.57	0.957
**2c**	45.29	0.935	38.93	0.961
**2d**	43.86	0.982	28.17	0.982
**2e**	96.25	0.955	128.33	0.961
**2f**	84.50	0.971	108.28	0.995
**2g**	121.57	0.934	100.43	0.982
**2h**	29.61	0.953	16.69	0.981
**2i**	**8.97**	**0.993**	**3.89**	**0.984**
**2j**	100.43	0.947	87.72	0.956
**2k**	77.00	0.997	72.94	0.981
**2l**	119.48	0.939	121.57	0.942
**4a**	57.27	0.962	39.82	0.987
**4b**	61.87	0.913	55.44	0.954
**4c**	76.15	0.934	63.00	0.924
**4d**	135.88	0.952	83.49	0.988
**4e**	111.77	0.937	92.40	0.995
**4f**	82.50	0.929	126.00	0.969
**4g**	80.58	0.971	78.75	0.950
**2m**	161.16	0.941	177.69	0.937
**2n**	141.42	0.976	147.44	0.979
**AZA**	69.30	0.998	59.74	0.998

a Values are expressed as mean ± SD of three independent experiments (*n* = 3). IC_50_ values were calculated from concentration inhibition curves. Statistical analysis was performed using one-way ANOVA, and differences among IC_50_ values were considered statistically significant at *p* < 0.05. AZA: acetazolamide (standard inhibitor).

**Scheme 8:**
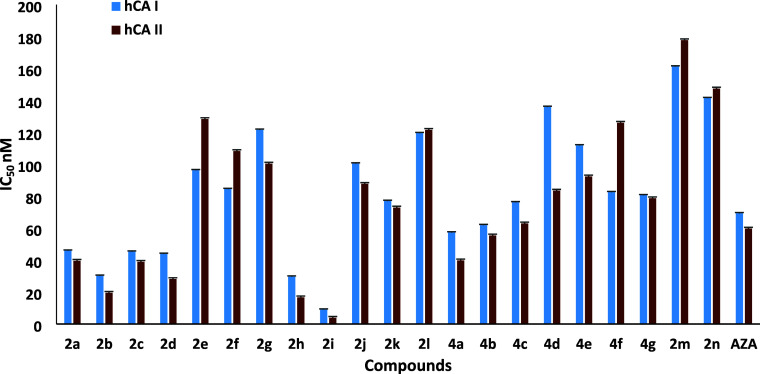
Inhibitory Activities (IC_50_, Mean ± SD) of Synthesized Sulfamate Derivatives against hCA I and hCA II

**Table 3:** Inhibitory Effects of Selected Compounds against hCA IX and hCA XII^a^

compounds	IC_50_ (nM) CA IX	*R* ^2^	CA XII	*R* ^2^
**2i**	6.45	0.991	5.12	0.988
**2h**	18.72	0.976	14.33	0.981
**2b**	26.85	0.968	21.47	0.972
AZA	25.40	0.995	18.60	0.994

a Values are expressed as mean ± SD of three independent experiments (*n* = 3). IC_50_ values were calculated from concentration–inhibition curves. Statistical analysis was performed using one-way ANOVA, and differences among IC_50_ values were considered statistically significant at *p* < 0.05. AZA: acetazolamide (standard inhibitor).

One-way ANOVA demonstrated that the differences among the IC_50_ values were statistically significant (*p* < 0.05).

The overall inhibition ranking for hCA I was as follows: **2i** (8.97 nM) > **2h** (29.61 nM) > **2b** (30.13 nM) > **2d** (43.86 nM) > **2c** (45.29 nM) > **2a** (45.89 nM) > **4a** (57.27 nM) > **4b** (61.87 nM) > AZA (69.30 nM) > **4c** (76.15 nM) > **2k** (77.00 nM) > **4g** (80.58 nM) > **4f** (82.50 nM) > **2f** (84.50 nM) > **2e** (96.25 nM) > **2j** (100.43 nM) > **4e** (111.77 nM) > **2l** (119.48 nM) > **2g** (121.57 nM) > **4d** (135.88 nM) > **2n** (141.42 nM) > **2m** (161.16 nM).

The inhibition ranking for hCA II was as follows: **2i** (3.89 nM) > **2h** (16.69 nM) > **2b** (19.57 nM) > **2d** (28.17 nM) > **2c** (38.93 nM) > **2a** (39.60 nM) > **4a** (39.82 nM) > **4b** (55.44 nM) > AZA (59.74 nM) > **4c** (63.00 nM) > **2k** (72.94 nM) > **4g** (78.75 nM) > **4d** (83.49 nM) > **2j** (87.72 nM) > **4e** (92.40 nM) > **2g** (100.43 nM) > **2f** (108.28 nM) > 2l (121.57 nM) > **4f** (126.00 nM) > **2e** (128.33 nM) > **2n** (147.44 nM) > **2m** (177.69 nM).

All compounds inhibited both isoenzymes at the nanomolar level, and the observed differences among the compounds were statistically significant (*p* < 0.05). Compound **2i** was identified as the most potent inhibitor for both isoenzymes, followed by compound **2h**. A similar inhibition trend was observed for hCA II, with compound **2i** exhibiting the lowest IC_50_ value. From a structure–activity relationship perspective, the strong inhibitory activity of compound **2i** was attributed to the presence of methoxy groups at the *meta* and *ortho* positions, which exerted a pronounced electron-donating effect and enhanced coordination of the sulfamate group to the Zn^+2^ center. In compound **2h**, which contains one methoxy group at the *para* position, the reduced electron-donating effect resulted in a decrease in activity; however, this compound still exhibited stronger inhibition than acetazolamide for both isoenzymes. In compounds **2b**, **2c**, and **2d**, replacement of the methoxy group with a methyl group weakened the electron-donating character, leading to a gradual reduction in inhibitory activity. In contrast, substitution with electron-withdrawing groups in compounds **2e**, **2f**, and **2g** resulted in lower inhibition than the standard drug for both isoenzymes. Halogen substituents (F, Br, and Cl) reduced electron density and weakened Zn^+2^ coordination. In compound **2j**, which has a nitro group at the meta position, inhibitory activity decreased further due to the strong electron-withdrawing effect of the nitro group, which limited effective binding of the sulfamate function. In compound **2l** (naphthalene), increased aromatic bulk led to reduced inhibitory activity, which was attributed to steric hindrance and limited accommodation within the enzyme active region. When the *p*-methyl compounds were examined, compounds **4b** and **4c** exhibited relatively high inhibitory activity compared to the standard drug against both hCA I and hCA II. The methyl substituent in these compounds provided sufficient electron density relative to the phenyl ring in compound **4a** and supported favorable hydrophobic interactions with the enzyme active region. In contrast, the electron-withdrawing halogen substituents in compounds **4d**, **4e**, and **4f** reduced the coordination of the sulfamate group to the Zn^+2^ center, leading to a significant loss of activity, particularly against hCA II. The thiophene ring in compound **4g** enabled hydrophobic interactions within the active region, preventing complete loss of activity; however, its limited electron-donating capacity resulted in only moderate inhibition. Finally, cinnamaldehyde derivatives **2m** and **2n** were among the weakest inhibitors of both isoenzymes, likely due to the absence of electronic modulation and directing interactions. Overall, the results demonstrated that electron-donating substituents enhanced carbonic anhydrase inhibition, whereas electron-withdrawing groups and bulky aromatic systems reduced inhibitory activity. These findings clearly indicate that both electronic effects and steric compatibility play a decisive role in determining the inhibition of carbonic anhydrase by sulfamate derivatives. To address isoform selectivity, the most potent compounds identified against hCA I and hCA II (**2b**, **2h**, and **2i**) were further evaluated against tumor-associated isoforms hCA IX and hCA XII (Table 3).

Compound **2i** exhibited the strongest inhibitory activity against both hCA IX and hCA XII, with IC_50_ values of 6.45 and 5.12 nM, respectively. Notably, these values are significantly lower than those of the reference inhibitor acetazolamide, indicating a pronounced affinity toward cancer-related isoforms. This result suggests that the electron-donating methoxy substituents in compound **2i** not only enhance Zn^+2^ coordination in hCA I and II but also favor binding within the more hydrophobic and structurally distinct active sites of CA IX and XII.^26^ Compound **2h** also demonstrated strong inhibition in the low-nanomolar range, although slightly weaker than that of **2i**. The presence of a single methoxy group appears sufficient to maintain favorable electronic interactions, but the reduced electron density compared to that of **2i** likely limits optimal binding efficiency. Compound **2b**, bearing methyl substituents, showed moderate inhibition against both isoforms. This trend is consistent with the previously observed structure–activity relationship, where weaker electron-donating groups result in reduced inhibitory potency.^27,28^ Importantly, comparison with acetazolamide revealed that compound **2i** surpassed the standard inhibitor against both CA IX and CA XII, whereas compounds **2h** and **2b** exhibited comparable or slightly improved activity. These findings indicate that while the synthesized sulfamate derivatives are potent inhibitors of hCA I and II, certain compounds, particularly **2i**, also display promising selectivity toward tumor-associated isoforms.

#### 2.2.1 Comparative Evaluation of Carbonic Anhydrase Inhibition with Literature Data

To provide a systematic and quantitative comparison with previously reported carbonic anhydrase inhibitors, the literature data together with the findings of the present study are summarized in Table 4. Supuran et al. demonstrated that the fundamental binding mechanism of sulfamate and sulfonamide derivatives to CA isoenzymes involves coordination to the Zn^+2^ ion and that this interaction is strongly influenced by the electronic properties of the ligand.^3,29^ In the present study, the observation that compounds bearing electron-donating substituents exhibit lower IC_50_ values is consistent with this established mechanistic framework. Compound **2i**, identified as the most potent inhibitor, displayed nanomolar-level inhibitory activity against both isoenzymes, which parallels the results reported by Congiu et al. for piperazinyl-ureidosulfamate derivatives. In that study, electron-donating groups positioned at the meta and *para* sites of the aromatic ring were shown to enhance sulfamate coordination to the Zn^+2^ center, resulting in a marked increase in inhibitory potency.^30^ Similarly, in compound **2i**, the presence of methoxy groups at the meta and *para* positions likely stabilizes Zn^+2^ coordination through a strong +M effect. This electronic contribution provides a plausible explanation for the superior inhibitory performance of compound **2i** compared with acetazolamide against both hCA I and hCA II. In addition, similar structure–activity relationships have been reported for α-sulfonoxy ketone derivatives synthesized via hypervalent iodine catalysis.^12^ Compounds bearing electron-donating substituents exhibited enhanced carbonic anhydrase inhibitory activity, as evidenced by molecular docking results indicating stronger interactions with the Zn^+2^-containing active site. These findings are consistent with the present results, where electron-donating groups improve the inhibitory potency of sulfamate derivatives. Moreover, docking analyses highlight the importance of both electronic effects and favorable binding orientation in stabilizing enzyme–inhibitor interactions, in agreement with the high activity observed for compound **2i**. Furthermore, Gerni et al.^4^ reported that celecoxib-derived pyrazole-linked sulfonamide derivatives effectively inhibit hCA I and hCA II, with docking studies indicating favorable interactions with the catalytic Zn^+2^ ion via hydrogen bonding and hydrophobic contacts. These findings are consistent with the present results, where sulfonamide/sulfamate-based compounds show strong inhibitory activity. In particular, the high potency of compound **2i** can be attributed to its favorable structural and electronic features that support optimal enzyme binding and effective coordination with the active site. For compound **2h**, which contains a single *para*-methoxy substituent, the observed reduction in inhibitory activity relative to **2i** are consistent with the electronic density activity relationship described by Aksu et al. (Table 4). Their study on dopamine-like sulfamide derivatives emphasized that both the number and positional distribution of electron-donating substituents on the aromatic ring directly affect inhibitory potency.^31^ The more limited electronic contribution of a single methoxy group reasonably accounts for the reduced activity of compound **2h**. This observation is consistent with the comparative data summarized in Table 3, where compounds bearing multiple electron-donating substituents generally exhibit stronger inhibition than monosubstituted analogs. The gradual decrease in inhibitory activity observed for compounds **2b**, **2c**, and **2d**, in which methoxy substituents were replaced by methyl groups, is consistent with findings reported by Daryadel et al. for menthol-derived sulfamates. It was noted that weak electron-donating groups, such as methyl substituents, can support Zn^+2^ coordination but generally confer lower inhibitory potency than substituents capable of strong resonance donation.^32^ The present results corroborate this trend. A pronounced reduction in inhibitory activity was observed for compounds **2e**, **2f**, and **2g** bearing electron-withdrawing substituents, a pattern frequently reported in the literature. The inductive electron-withdrawing effects of halogen atoms weaken coordination between the sulfamate group and the Zn^+2^ ion, leading to diminished inhibitory efficacy.^3,33^ In particular, the low activity of compound **2j**, containing a nitro substituent, can be attributed to the strong −M and −I effects of the nitro group, which significantly limit the binding capability of the sulfamate moiety. This observation is consistent with the loss of activity reported for nitro-substituted CA inhibitors by Supuran and Scozzafava. The reduced inhibitory activity observed for compounds **2k** (thiophene) and **2l** (naphthalene), which possess increased aromatic volume, highlights the importance of steric factors. Previous studies have shown that bulky and rigid aromatic systems can hinder optimal accommodation within the enzyme active site, thereby negatively affecting binding geometry.^29^ These findings indicate that steric compatibility is at least as critical as electronic effects in determining inhibitory potency. The activity profiles of the *p*-methyl compounds also showed substantial agreement with the literature. Compounds **4b** and **4c** exhibited stronger inhibitory effects than acetazolamide against both hCA I and hCA II, which can be attributed to the favorable electronic density and hydrophobic interactions imparted by methyl substituents on the aromatic ring. In contrast, the electron-withdrawing halogen substituents present in compounds **4d**, **4e**, and **4f** weakened Zn^+2^ coordination, resulting in a significant loss of activity, particularly against hCA II. Compound **4g**, containing a thiophene ring, did not become completely inactive due to residual hydrophobic interactions; however, its limited electron-donating capacity was insufficient to confer strong inhibition. Similar trends have been reported for heterocyclic sulfamate derivatives.^30^ In addition to the widely studied hCA I and hCA II isoenzymes, tumor-associated carbonic anhydrase isoforms hCA IX and hCA XII have gained considerable attention due to their overexpression in hypoxic tumors and their critical role in cancer progression. Recent studies have demonstrated that selective inhibition of these isoforms represents a promising therapeutic strategy for anticancer drug development. For instance, benzenesulfonamide- and sulfamate-based inhibitors have been reported to exhibit strong inhibitory activity against hCA IX and XII, often in the low-nanomolar range.^9,30^ In the present study, the inhibitory activities of selected compounds (**2i**, **2h**, and **2b**) against hCA IX and hCA XII were consistent with reports in the literature. In particular, compound **2i** exhibited superior inhibition compared to acetazolamide, suggesting that the propargyl sulfamate scaffold can effectively target tumor-associated CA isoforms. This finding aligns with previous reports indicating that electron-donating substituents enhance binding affinity and may contribute to improved selectivity toward cancer-related isoforms.^26–28^

**Table 4:** Comparative Evaluation of Carbonic Anhydrase Inhibitors from the Literature and the Present Study

compound/class	target isoenzyme	IC_50_ (nM)	reference
propargyl sulfamate (**2i**)	hCA I/II	8.97/3.89	this study
propargyl sulfamate (**2h**)	hCA I/II	29.61/16.69	this study
acetazolamide (AZA)	hCA I/II	69.30/59.74	standard
piperazinyl-ureidosulfamate	hCA I/II	9.40–896.80/1.00–94.40	^27^
dopamine-derived sulfamides	hCA I/II	61.00–1822/1.47–2.95	^29^
menthol-derived sulfamates	hCA I/II	41.82–53.55/15.97–29.96	^15^
pyrazole-linked sulfonamide	hCA I/II	10.76–2566/7.96–103.83	^4^
α-sulfonoxy ketones	hCA I/II	207490–641070/310760–529410	^12^
propargyl sulfamate (**2i**)	hCA XI/XII	6.45/5.12	this study
propargyl sulfamate (**2h**)	hCA XI/XII	18.72/14.33	this study
acetazolamide (AZA)	hCA IX/XII	25.40/18.60	standard
piperazinyl-ureidosulfamate	hCA IX/XII	5.4–73.6/4.8–65.2	^26^
benzenesulfonamides	hCA IX/XII	6.2–58.4/5.8–62.7	^9^

Overall, the consistency between the present findings and previously reported studies summarized in Table 4 confirms that both electronic and steric factors are key determinants in carbonic anhydrase inhibition.

## 3 Conclusions

In this study, a practical and efficient one-step methodology was developed for the synthesis of novel propargyl sulfamate derivatives from propargyl alcohols using CSI under catalyst-free conditions. The reaction proceeded without the use of Lewis acids or preactivation of hydroxyl groups, thereby overcoming several limitations commonly associated with conventional synthetic approaches. A plausible reaction mechanism was proposed to rationalize the observed reactivity and product formation. The synthesized propargyl sulfamate derivatives were subsequently evaluated for their in vitro inhibitory activities against hCA I and hCA II. The compounds exhibited inhibitory effects in the nanomolar range, indicating a strong affinity toward both isoenzymes. Among the tested derivatives, compound **2i** displayed the most pronounced inhibition, surpassing the reference inhibitor in terms of potency against both hCA I and hCA II. Compound **2i** is 7.72 times more active than the standard drug AZA in inhibiting the hCA I isoform and 15.35 times more active in inhibiting the hCA II isoform. Furthermore, the most active compounds were also evaluated against tumor-associated isoforms hCA IX and hCA XII. In line with the results obtained for hCA I and hCA II, compound **2i** exhibited superior inhibitory activity compared to acetazolamide, demonstrating approximately 3.9-fold higher potency against hCA IX and 3.6-fold higher potency against hCA XII. These findings suggest that compound **2i**, owing to its potent carbonic anhydrase inhibitory activity, is a promising candidate for further drug development. Overall, the combined synthetic accessibility and notable biological activity of these propargyl sulfamate derivatives highlight the potential of this methodology for developing new carbonic anhydrase inhibitors and support further investigations into their structure–activity relationships and therapeutic relevance. Moreover, this methodology could serve as an efficient strategy for synthesizing cinnamaldehyde derivatives.

## 4 Experimental Section

### 4.1 General Procedure for Synthesis of Propargyl Alcohols (**1a**–**l**, **3a**–**g**)

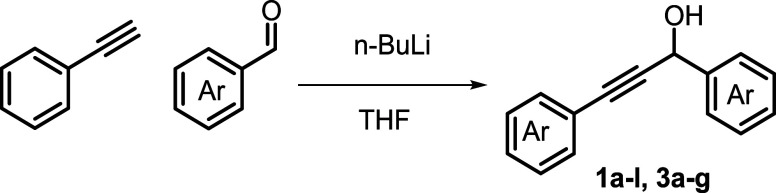



To a solution of phenylacetylene (1.0 mol) in fresh THF (30 mL) was added *n*-BuLi (1.2 mol) under N_2_ gas at −78 °C. After the mixture was stirred at the same temperature for 15 min, benzaldehyde derivatives were added to the reaction mixture at −78 °C. The reaction was stirred at room temperature for 4 h and was stopped. The mixture was extracted with EtOAc (2 × 30 mL). The combined extracts were dried over Na_2_SO_4_, and the solvent was removed in the evaporator. The product was obtained in pure form.

### 4.2 General Procedure for Synthesis of Propargyl Sulfamate (**2a**–**l**, **4a**–**g**)

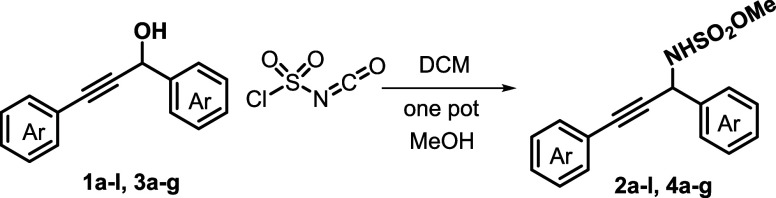



To a solution of propargyl alcohols (1.0 mol) in CH_2_CI_2_ (15 mL) was added slowly chlorosulfonyl isocyanate (1.1 mol) over 2 min at 0 °C. The reaction mixture was stirred at room temperature for 2 h. The reaction mixture was then cooled to 0 °C, and MeOH (1 mL) was added and stirred more for 15 min. The mixture was extracted with CH_2_CI_2_ (2 × 20 mL). The combined extracts were dried over Na_2_SO_4_, and the solvent was removed in the evaporator. The products were purified by thin-layer chromatography.

### 4.3 Synthesis of Methyl (1,3-Diphenylprop-2-yn-1-yl)­sulfamate (**2a**)

^1^H NMR (400 MHz, CDCl_3_) δ 7.63 (d, *J* = 7.8, Hz, 2H), 7.49 (dd, *J* = 7.4, 2.1 Hz, 2H), 7.45–7.33 (m, 6H), 5.60 (d, *J* = 8.3 Hz, 1H), 5.15 (d, *J* = 8.3 Hz, 1H), 3.84 (s, 3H). ^13^C NMR (100 MHz, CDCl_3_) δ 137.49, 131.96, 129.20, 129.16, 129.09, 128.69, 127.59, 122.14, 86.90, 85.88, 57.02 (CHN), 50.64 (OMe). HR-MS: *m*/*z* calc. For C_16_H_15_NO_3_S: 301.0773; Found: 301.0787.

### 4.4 Synthesis of Methyl (3-Phenyl-1-(*o*-tolyl)­prop-2-yn-1-yl)­sulfamate (**2b**)

^1^H NMR (400 MHz, CDCl_3_) δ 7.70 (dd, *J* = 6.9, 2.1 Hz, 1H), 7.50–7.44 (m, 2H), 7.40–7.31 (m, 3H), 7.30–7.21 (m, 3H), 5.73 (d, *J* = 8.0 Hz, 1H), 4.97 (d, *J* = 7.9 Hz, 1H), 3.85 (s, 3H), 2.51 (s, 3H). ^13^C NMR (100 MHz, CDCl_3_) δ 136.56, 135.39, 131.91, 131.42, 129.30, 129.11, 128.68, 127.72, 126.76, 122.27, 86.57, 86.23, 57.03, 48.29, 19.29. HR-MS: *m*/*z* (M+Na) calc. For: C_17_H_17_NNaO_3_S: 338.0827; Found: 338.0802.

### 4.5 Synthesis of Methyl (3-Phenyl-1-(*m*-tolyl)­prop-2-yn-1-yl)­sulfamate (**2c**)

^1^H NMR (400 MHz, CDCl_3_) δ 7.53–7.46 (m, 2H), 7.45–7.24 (m, 6H), 7.19 (d, *J* = 7.5 Hz, 1H), 5.57 (d, *J* = 8.2 Hz, 1H), 5.05 (d, *J* = 8.2 Hz, 1H), 3.87 (s, 3H), 2.40 (s, 3H). ^13^C NMR (100 MHz, CDCl_3_) δ 139.02, 137.37, 131.95, 129.85, 129.15, 129.05, 128.67, 128.23, 124.56, 122.21, 86.74, 85.97, 57.02, 50.62, 21.67. HR-MS: *m*/*z* (M+Na) calc. For: C_17_H_17_NNaO_3_S: 338.0827; Found: 338.0808.

### 4.6 Synthesis of Methyl (3-Phenyl-1-(*p*-tolyl)­prop-2-yn-1-yl)­sulfamate (**2d**)

^1^H NMR (400 MHz, CDCl_3_) δ 7.53–7.47 (m, 2H), 7.45–7.29 (m, 6H), 7.19 (d, *J* = 7.5 Hz, 1H), 5.57 (d, *J* = 8.2 Hz, 1H), 5.05 (d, *J* = 8.2 Hz, NH, 1H), 3.87 (s, 3H), 2.40 (s, 3H). ^13^C NMR (100 MHz, CDCl_3_) δ 138.97, 134.61, 131.95, 129.79, 129.12, 128.67, 127.51, 122.25, 86.60, 86.15, 56.98 (CHN), 50.37 (OMe), 21.40. HR-MS: *m*/*z* (M+Na) calc. For: C_17_H_17_NNaO_3_S: 338.0827; Found: 338.0821.

### 4.7 Synthesis of Methyl (1-(4-Fluorophenyl)-3-phenylprop-2-yn-1-yl)­sulfamate (**2e**)

^1^H NMR (400 MHz, CDCl_3_) δ 7.63–7.57 (m, 2H), 7.49 (dd, *J* = 7.6, 1.8 Hz, 2H), 7.42–7.31 (m, 3H), 7.08 (t, *J* = 8.6 Hz, 2H), 5.59 (d, *J* = 8.5 Hz, 1H), 5.56 (d, *J* = 8.5 Hz, 1H), 3.82 (s, 3H). ^13^C NMR (100 MHz, CDCl_3_) δ 163.05 (d, *J*_C,F_^1^ = 247.9 Hz), 133.49 (d, *J*_C,F_^4^ = 3.1 Hz), 131.98, 129.53 (d, *J*_C,F_^3^ = 8.4 Hz), 129.33, 128.76, 122.01, 115.94 (d, *J*_C,F_^2^ = 11.4 Hz), 87.07, 85.68, 57.05, 49.90. HR-MS: *m*/*z* (M) calc. For: C_16_H_14_FNO_3_S: 319.0678; Found: 319.0681.

### 4.8 Synthesis of Methyl (1-(4-Chlorophenyl)-3-phenylprop-2-yn-1-yl)­sulfamate (**2f**)

^1^H NMR (400 MHz, CDCl_3_) δ 7.57–7.53 (m, 2H), 7.50–7.46 (m, 2H), 7.39–7.33 (m, 5H), 5.60 (d, *J* = 8.5 Hz, 1H), 5.54 (d, *J* = 8.5 Hz, 1H), 3.82 (s, 3H). ^13^C NMR (100 MHz, CDCl_3_) δ 136.15, 134.92, 131.99, 129.37, 129.25, 129.04, 128.76, 121.92, 87.18, 85.42, 57.08, 49.93. HR-MS: *m*/*z* (M) calc. For: C_16_H_14_ClNO_3_S: 335.0383; Found: 335.0386.

### 4.9 Synthesis of Methyl (1-(4-Bromophenyl)-3-phenylprop-2-yn-1-yl)­sulfamate (**2g**)

^1^H NMR (400 MHz, CDCl_3_) δ 7.63–7.40 (m, 6H), 7.40–7.29 (m, 3H), 5.54 (d, *J* = 8.4 Hz, 1H), 5.42 (d, *J* = 8.5 Hz, 1H), 3.83 (s, 3H). ^13^C NMR (100 MHz, CDCl_3_) δ 136.63, 132.23, 131.98, 129.38, 129.32, 128.75, 123.15, 121.86, 87.24, 85.30, 57.09, 50.03. HR-MS: *m*/*z* (M) calc. For: C_16_H_14_BrNO_3_S: 378.9878; Found: 378.9876.

### 4.10 Synthesis of Methyl (1-(4-Methoxyphenyl)-3-phenylprop-2-yn-1-yl)­sulfamate (**2h**)

^1^H NMR (400 MHz, CDCl_3_) δ 7.53 (d, *J* = 8.6 Hz, 2H), 7.49–7.46 (m, 2H), 7.41–7.31 (m, 3H), 6.92 (d, *J* = 8.7 Hz, 2H), 5.54 (d, J = 8.2 Hz, 1H), 5.29 (d, *J* = 8.2 Hz, 1H), 3.83 (s, 3H), 3.81 (s, 3H). ^13^C NMR (100 MHz, CDCl_3_) δ 160.14, 131.94, 129.65, 129.13, 128.93, 128.68, 122.24, 114.45, 86.64, 86.22, 56.96, 55.60, 50.15. HR-MS: *m*/*z* (M) calc. For: C_17_H_17_NO_4_S: 331.0878; Found: 331.0881.

### 4.11 Synthesis of Methyl (1-(2,5-Dimethoxyphenyl)-3-phenylprop-2-yn-1-yl)­sulfamate (**2i**)

^1^H NMR (400 MHz, CDCl_3_) δ 7.45–7.37 (m, 2H), 7.33–7.25 (m, 3H), 7.07 (d, *J* = 2.3 Hz, 1H), 6.87 (d, *J* = 2.7 Hz, 2H), 5.77 (d, *J* = 8.3 Hz, 1H), 5.63 (d, *J* = 8.6 Hz, 1H), 3.88 (s, 3H), 3.79 (s, 3H), 3.78 (s, 3H). ^13^C NMR (100 MHz, CDCl_3_) δ 153.93, 151.09, 131.92, 128.89, 128.57, 127.06, 122.50, 115.11, 114.75, 113.02, 86.44, 85.21, 56.87, 56.65, 56.03, 47.47. HR-MS: *m*/*z* (M+Na) calc. For: C_18_H_19_NNaO_5_S: 384.0882; Found: 384.0880.

### 4.12 Synthesis of Methyl (1-(3-Nitrophenyl)-3-phenylprop-2-yn-1-yl)­sulfamate (**2j**)

^1^H NMR (400 MHz, CDCl_3_) δ 8.50 (s, 1H), 8.24–8.18 (m, 1H), 7.99–7.94 (m, 1H), 7.59 (t, *J* = 8.0 Hz, 1H), 7.52–7.45 (m, 2H), 7.42–7.30 (m, 3H), 5.79 (d, *J* = 8.5 Hz, 1H), 5.67 (d, *J* = 8.5 Hz, 1H), 3.88 (s, 3H). ^13^C NMR (100 MHz, CDCl_3_) δ 148.65, 139.88, 133.84, 132.03, 130.18, 129.60, 128.79, 123.93, 122.54, 121.49, 88.01, 84.48, 57.26, 49.83. HR-MS: *m*/*z* (M) calc. For: C_16_H_14_N_2_O_5_S: 346.0623; Found: 346.0624.

### 4.13 Synthesis of Methyl (3-Phenyl-1-(thiophen-2-yl)­prop-2-yn-1-yl)­sulfamate (**2k**)

^1^H NMR (400 MHz, CDCl_3_) δ 7.52–7.47 (m, 2H), 7.39–7.32 (m, 4H), 7.29 (d, *J* = 3.5 Hz, 1H), 7.01 (dd, *J* = 5.1, 3.6 Hz, 1H), 5.81 (d, *J* = 8.5 Hz, 1H), 5.20 (s, 1H), 3.88 (s, 3H). ^13^C NMR (100 MHz, CDCl_3_) δ 141.08 (s), 132.02, 129.37, 128.70, 127.32, 127.09, 126.85, 121.83, 86.11, 85.52, 57.19, 46.45. HR-MS: *m*/*z* (M) calc. For: C_14_H_13_NO_3_S_2_: 307.0337; Found: 307.0335.

### 4.14 Synthesis of Methyl (1-(Naphthalen-2-yl)-3-phenylprop-2-yn-1-yl)­sulfamate (**2l**)

^1^H NMR (400 MHz, CDCl_3_) δ 8.08 (s, 1H), 7.93–7.83 (m, 3H), 7.69 (dd, *J* = 8.6, 1.8 Hz, 1H), 7.56–7.49 (m, 4H), 7.42–7.33 (m, 3H), 5.76 (d, *J* = 8.3 Hz, 1H), 5.25 (d, *J* = 8.3 Hz, 1H), 3.85 (s, 3H). ^13^C NMR (100 MHz, CDCl_3_) δ 134.72, 133.52, 133.30, 132.00, 129.24, 129.21, 128.72, 128.47, 127.95, 126.98, 126.87, 126.71, 125.06, 122.15, 87.16, 85.88, 57.07, 50.83. HR-MS: *m*/*z* (M) calc. For: C_20_H_17_NO_3_S: 351.0929; Found: 351.0933.

### 4.15 Synthesis of Methyl (1-Phenyl-3-(*p*-tolyl)­prop-2-yn-1-yl)­sulfamate (**4a**)

^1^H NMR (400 MHz, CDCl_3_) δ 7.63 (d, *J* = 7.3 Hz, 2H), 7.45–7.39 (m, 3H), 7.38 (d, *J* = 8.0 Hz, 2H), 7.15 (d, *J* = 8.0 Hz, 2H), 5.59 (d, *J* = 8.4 Hz, 1H), 5.13 (d, *J* = 8.3 Hz, 1H), 3.84 (s, 3H), 2.37 (s, 3H). ^13^C NMR (100 MHz, CDCl_3_) δ 139.46, 137.55, 131.86, 129.45, 129.14, 129.06, 127.61, 119.02, 87.06, 85.14, 57.04, 50.65, 21.78. HR-MS: *m*/*z* (M+Na) calc. For: C_17_H_17_NNaO_3_S: 338.0827; Found: 338.0830.

### 4.16 Synthesis of Methyl (1-(*o*-Tolyl)-3-(*p*-tolyl)­prop-2-yn-1-yl)­sulfamate (**4b**)

^1^H NMR (400 MHz, CDCl_3_) δ 7.70 (dd, *J* = 7.0, 2.0 Hz, 1H), 7.36 (d, *J* = 8.1 Hz, 2H), 7.30–7.20 (m, 3H), 7.14 (d, *J* = 8.0 Hz, 2H), 5.72 (d, *J* = 8.0 Hz, 1H), 4.99 (d, *J* = 8.0 Hz, 1H), 3.85 (s, 3H), 2.50 (s, 3H), 2.36 (s, 3H). ^13^C NMR (100 MHz, CDCl_3_) δ 139.34, 136.57, 135.49, 131.80, 131.39, 129.43, 129.24, 127.73, 126.73, 119.16, 86.72, 85.52, 57.02, 48.30, 21.76, 19.30. HR-MS: *m*/*z* (M+Na) calc. For: C_18_H_19_NNaO_3_S: 352.0983; Found: 352.0968.

### 4.17 Synthesis of Methyl (1-(*m*-Tolyl)-3-(*p*-tolyl)­prop-2-yn-1-yl)­sulfamate (**4c**)

^1^H NMR (400 MHz, CDCl_3_) δ 7.46–7.34 (m, 4H), 7.30 (t, *J* = 8.0 Hz, 1H), 7.16 (t, *J* = 8.8 Hz, 3H), 5.55 (d, *J* = 8.3 Hz, 1H), 5.10 (d, *J* = 8.3 Hz, 1H), 3.85 (s, 3H), 2.39 (s, 3H), 2.37 (s, 3H). ^13^C NMR (100 MHz, CDCl_3_) δ 139.40, 138.97, 137.48, 131.85, 129.79, 129.43, 129.02, 128.25, 124.58, 119.11, 86.90, 85.33, 57.02, 50.62, 21.76, 21.68. HR-MS: *m*/*z* (M+Na) calc. For: C_18_H_19_NNaO_3_S: 352.0983; Found: 352.0972.

### 4.18 Synthesis of Methyl (1-(4-Chlorophenyl)-3-(*p*-tolyl)­prop-2-yn-1-yl)­sulfamate (**4d**)

^1^H NMR (400 MHz, CDCl_3_) δ 7.55 (d, *J* = 8.7 Hz, 2H), 7.39–7.34 (m, 4H), 7.15 (d, *J* = 7.9 Hz, 2H), 5.55 (d, *J* = 8.4 Hz, 1H), 5.23 (d, *J* = 8.4 Hz, 1H), 3.84 (s, 3H), 2.37 (s, 3H). ^13^C NMR (100 MHz, CDCl_3_) δ 139.67, 136.15, 134.95, 131.86, 129.49, 129.24, 129.02, 118.73, 87.43, 84.63, 57.09, 50.02, 21.78. HR-MS: *m*/*z* (M) calc. For: C_17_H_16_ClNO_3_S: 349.0539; Found: 349.0544.

### 4.19 Synthesis of Methyl (1-(4-Fluorophenyl)-3-(*p*-tolyl)­prop-2-yn-1-yl)­sulfamate (**4e**)

^1^H NMR (400 MHz, CDCl_3_) δ 7.60 (dd, *J* = 8.7, 5.3 Hz, 2H), 7.37 (d, *J* = 8.0 Hz, 2H), 7.15 (d, *J* = 8.0 Hz, 2H), 7.08 (t, *J* = 8.6 Hz, 2H), 5.56 (d, *J* = 8.4 Hz, 1H), 5.34 (d, *J* = 8.4 Hz, 1H), 3.83 (s, 3H), 2.37 (s, 3H). ^13^C NMR (100 MHz, CDCl_3_) δ 163.03 (d, *J*_C,F_^1^ = 248.0 Hz), 139.60 (s), 133.51 (d, *J*_C,F_^4^ = 3.2 Hz), 131.85 (s), 129.51 (d, *J*_C,F_^3^ = 8.3 Hz), 129.48 (s), 118.84 (s), 115.98 (d, *J*_C,F_^2^ = 21.8 Hz), 87.28, 84.91, 57.04, 49.96, 21.77. HR-MS: *m*/*z* (M) calc. For: C_17_H_16_FNO_3_S: 333.0835; Found: 333.0827.

### 4.20 Synthesis of Methyl (1-(4-Bromophenyl)-3-(*p*-tolyl)­prop-2-yn-1-yl)­sulfamate (**4f**)

^1^H NMR (400 MHz, CDCl_3_) δ 7.53 (d, *J* = 8.6 Hz, 2H), 7.49 (d, *J* = 8.7 Hz, 2H), 7.36 (d, *J* = 8.1 Hz, 2H), 7.15 (d, *J* = 8.0 Hz, 2H), 5.54 (d, *J* = 8.4 Hz, 1H), 5.18 (d, *J* = 8.4 Hz, 1H), 3.85 (s, 3H), 2.37 (s, 3H). ^13^C NMR (100 MHz, CDCl_3_) δ 139.68, 136.68, 132.22, 131.85, 129.49, 129.32, 123.14, 118.71, 87.49, 84.56, 57.10, 50.10, 21.86. HR-MS: *m*/*z* (M) calc. For: C_17_H_16_BrNO_3_S: 393.0034; Found: 393.0047.

### 4.21 Synthesis of Methyl (1-(Thiophen-2-yl)-3-(*p*-tolyl)­prop-2-yn-1-yl)­sulfamate (**4g**)

^1^H NMR (400 MHz, CDCl_3_) δ 7.38 (d, *J* = 8.1 Hz, 2H), 7.33 (dd, *J* = 5.1, 1.2 Hz, 1H), 7.29–7.26 (m, 1H), 7.15 (d, *J* = 8.3 Hz, 2H), 7.00 (dd, *J* = 5.1, 3.6 Hz, 1H), 5.79 (d, *J* = 8.5 Hz, 1H), 5.26 (d, *J* = 8.5 Hz, 1H), 3.87 (s, 3H), 2.37 (s, 3H). ^13^C NMR (100 MHz, CDCl_3_) δ 141.27, 139.64, 131.92, 129.46, 127.30, 127.05, 126.80, 118.74, 86.28, 84.88, 57.18, 46.46, 21.79. HR-MS: *m*/*z* (M+Na) calc. For: C_15_H_15_NNaO_3_S_2_: 344.0391; Found: 344.0396.

### 4.22 Reaction of (*E*)-1,5-Diphenylpent-1-en-4-yn-3-ol with CSI

#### 4.22.1 First Isomer (**2m**)

^1^H NMR (400 MHz, CDCl_3_) δ 7.97–7.00 (m, 10H), 6.35 (dd, *J* = 15.8, 6.4 Hz, 1H), 5.97 (dd, *J* = 15.8, 1.5 Hz, 1H), 5.16 (t, *J* = 6.9 Hz, 1H), 4.85 (d, *J* = 7.1 Hz, 1H), 3.72 (s, 3H). ^13^C NMR (100 MHz, CDCl_3_) δ 140.66, 138.81, 131.78, 129.38, 128.88, 128.73, 128.58, 127.37, 123.04, 113.28, 91.82, 86.73, 60.13, 56.84. HR-MS: *m*/*z* (M+Na) calc. For: C_18_H_17_NNaO_3_S: 350.0827; Found: 350.0829.

#### 4.22.2 Second Isomer (**2n**)

^1^H NMR (400 MHz, CDCl_3_) δ 7.51–7.31 (m, 10H), 6.11 (dd, *J* = 10.6, 8.7 Hz, 1H), 5.94 (dd, *J* = 10.7, 0.6 Hz, 1H), 5.70 (dd, *J* = 8.7, 6.4 Hz, 1H), 5.04 (d, *J* = 6.4 Hz, 1H), 3.77 (s, 3H).

### 4.23 Inhibition Assay of Carbonic Anhydrase Enzymes

The inhibitory effects of the sulfamate derivatives on hCA IX, hXII, hCA I, and hCA II were evaluated using the esterase activity assay, following a well-established spectrophotometric method. In this assay, *p*-nitrophenyl acetate (*p*-NPA) was employed as the substrate. The method is based on the enzymatic hydrolysis of *p*-NPA by carbonic anhydrase to yield *p*-nitrophenol and acetic acid. The formation of *p*-nitrophenol was monitored spectrophotometrically by measuring the increase in absorbance at 348 nm at 25 °C using a UV–vis spectrophotometer. Enzyme activity was calculated using the molar extinction coefficient of *p*-nitrophenol at 348 nm (ε = 5.4 × 10^3^ M^–1^ cm^–1^). The inhibitory activities of compound sulfamate against hCA I and hCA II were determined by measuring enzyme activity at a minimum of five different inhibitor concentrations. For each inhibitor concentration, enzyme activity was expressed as a percentage of residual activity, calculated relative to inhibitor-free control assays, which were defined as 100% activity. The reference inhibitor acetazolamide was evaluated under identical experimental conditions.^34^ Dose–response curves were constructed by plotting residual enzyme activity (%) against inhibitor concentration (nM). IC_50_ values, defined as the inhibitor concentration required to reduce enzyme activity to 50% of the control value, were calculated from these plots for both hCA I and hCA II isoenzymes independently.^35^

### 4.24 Statistical Analysis

All experiments performed in the study were repeated three times for each sample. The results are presented as means ± SDs (*n* = 3). One-way ANOVA was used, followed by Tukey’s post hoc test.

## Supplementary Material



## Notes

^1^H and ^13^C NMR spectra of synthesized compounds (PDF)
