# Supplementary material for: Efficient Synthesis of Novel Propargyl Sulfamate Derivatives and Their Potent Inhibitory Effects on Human Carbonic Anhydrase Isoenzymes
Source: ACS Omega. 2026 Jun 18;11(25):37419–29. doi: 10.1021/acsomega.6c02129 (PMC13325173; doi:10.1021/acsomega.6c02129)

Efficient Synthesis of Novel Propargyl Sulfamate Derivatives and Their Potent Inhibitory Effects on Human Carbonic Anhydrase Isoenzymes

Ufuk Atmaca[1*], Songul Bayrak[1], Cetin Bayrak[1,2*]

[1]Department of Chemistry, Faculty of Science, Ataturk University, Erzurum 25240, Turkey

[2]Dogubayazit Ahmed-i Hani Vocational School, Agri Ibrahim Cecen University, Agri 04400, Turkey

Abstract: In the present study, a total of 21 novel propargyl sulfamate derivatives were designed and synthesized as potential inhibitors of human carbonic anhydrase I and II (hCA I and hCA II). An efficient and practical synthetic protocol was developed based on the reaction of propargyl alcohols with chlorosulfonyl isocyanate (CSI), affording the target sulfamate compounds in good yields. The inhibitory activities of all synthesized compounds against hCA I and hCA II isoenzymes were evaluated in vitro. The compounds exhibited potent inhibition, with IC_{50} values ranging from 8.97 to 161.16 nM for hCA I and from 3.89 to 177.69 nM for hCA II. Among the tested molecules, Methyl (1-(2,5-dimethoxyphenyl)-3-phenylprop-2-yn-1-yl)sulfamate (**2i)** emerged as the most active derivative, displaying superior inhibitory activity compared to the reference drug acetazolamide (AZA), with IC_{50} values of 8.97 nM and 3.89 nM against hCA I and hCA II, respectively. Compound **2i** is **7.72** times more active than the standard drug AZA in inhibiting the hCAI isoform and **15.35** times more active in inhibiting the hCAII isoform. Furthermore, selected compounds (**2i**, **2h**, and **2b**) were evaluated against tumor-associated isoenzymes hCA IX and hCA XII, exhibiting strong inhibition with IC_{50} values ranging from 5.12 to 26.85 nM. Notably, compound 2i demonstrated the highest activity with IC_{50} values of 6.45 nM for hCA IX and 5.12 nM for hCA XII, surpassing the reference inhibitor AZA. These findings indicated that **2i**, because of its strong carbonic anhydrase inhibition effect, represented a promising candidate for further drug development. This methodology may also serve as a convenient strategy for generating cinnamaldehyde derivatives.

Keywords: Propargyl sulfamate, Chlorosulfonyl isocyanate, Carbonic anhydrase and Enzyme inhibition

^{1}H and ^{13}C-NMR spectra of synthesized compounds

Compound 2a

^{1}H-NMR Spectrum of the (400 MHz, $CDCl_3$).

^{13}C-NMR Spectrum of the (100 MHz, $CDCl_3$).

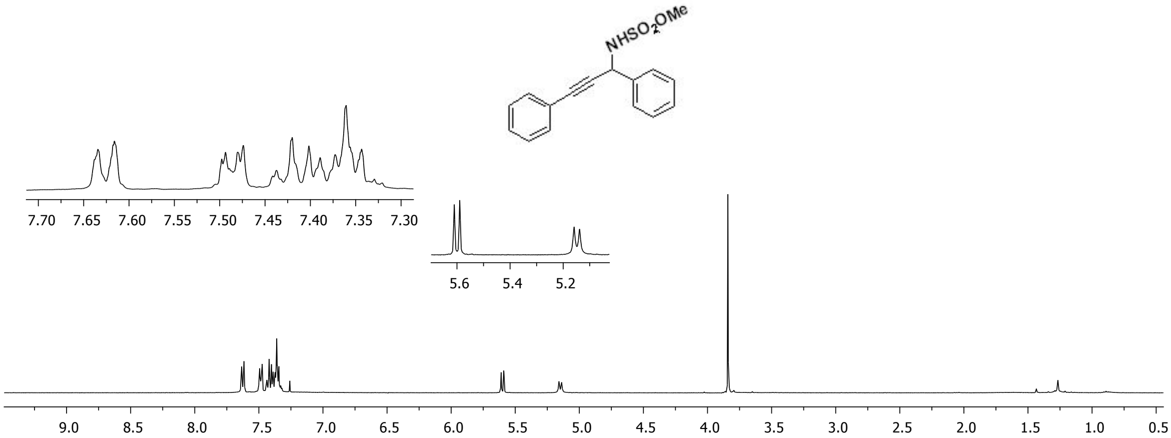

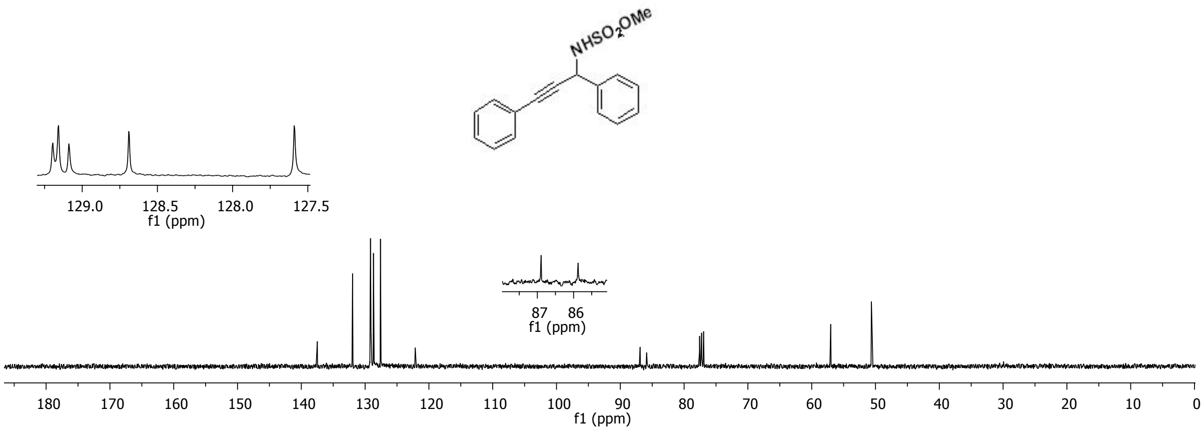

Compound 2b

^{1}H-NMR Spectrum of the (400 MHz, $CDCl_3$).

^{13}C-NMR Spectrum of the (100 MHz, $CDCl_3$).

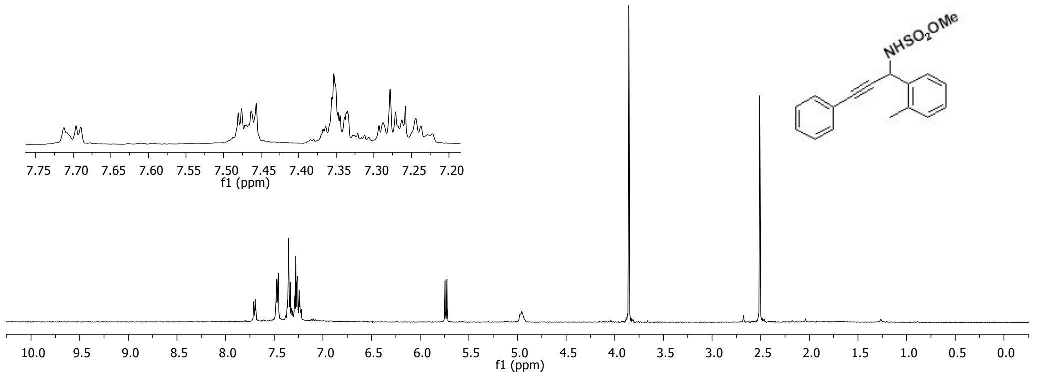

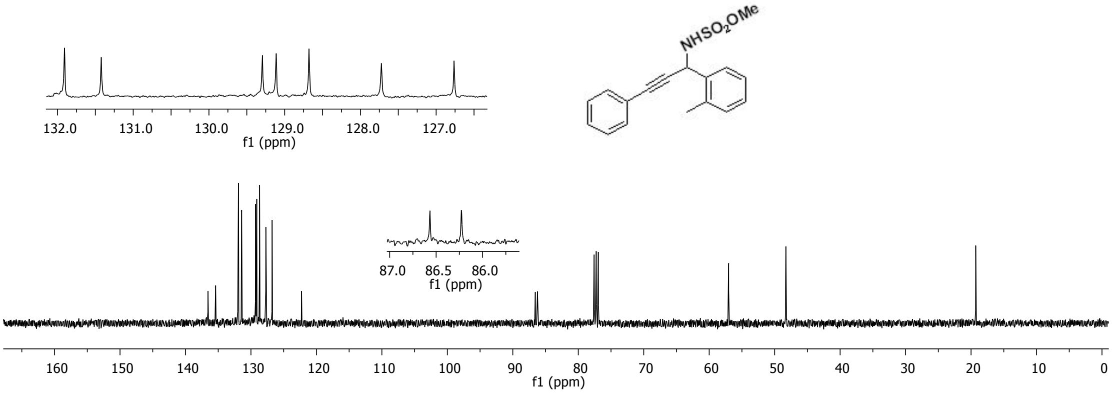

Compound 2c

^{1}H-NMR Spectrum of the (400 MHz, $CDCl_3$).

^{13}C-NMR Spectrum of the (100 MHz, $CDCl_3$).

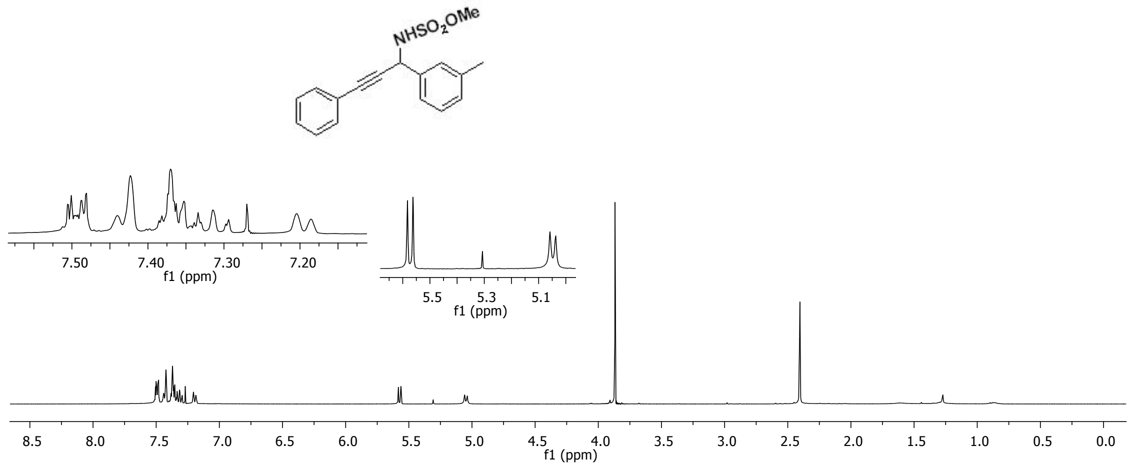

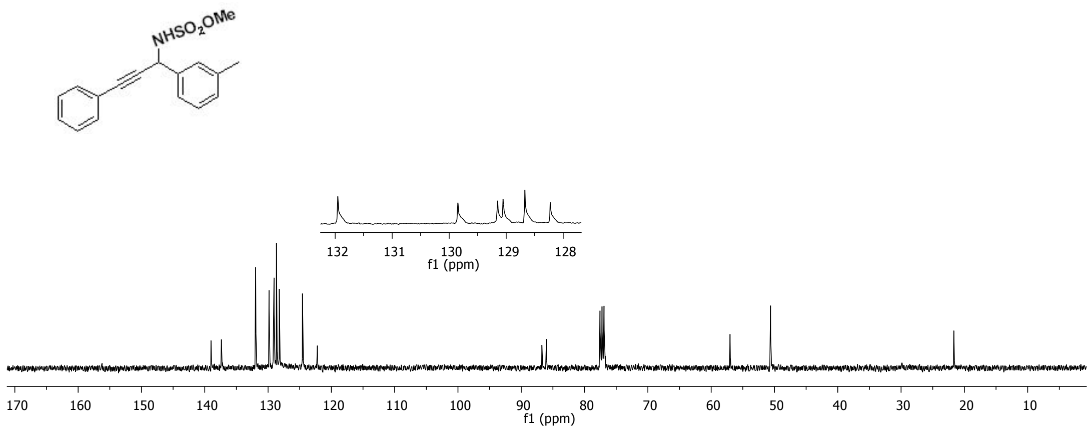

Compound 2d

^{1}H-NMR Spectrum of the (400 MHz, $CDCl_3$).

^{13}C-NMR Spectrum of the (100 MHz, $CDCl_3$).

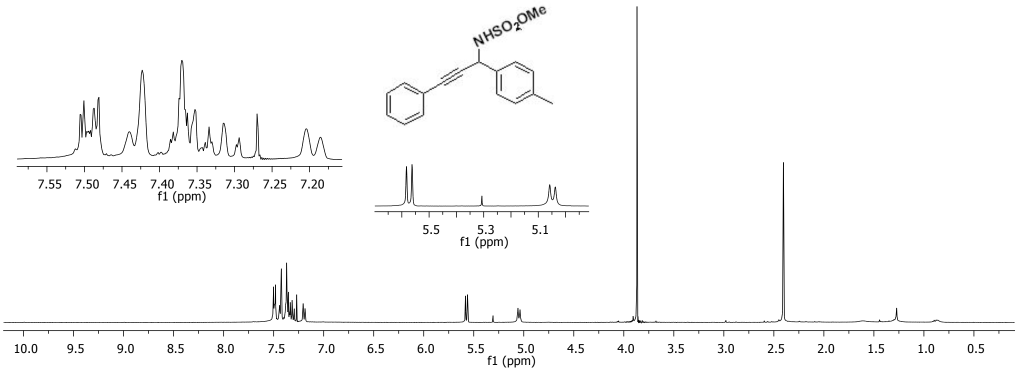

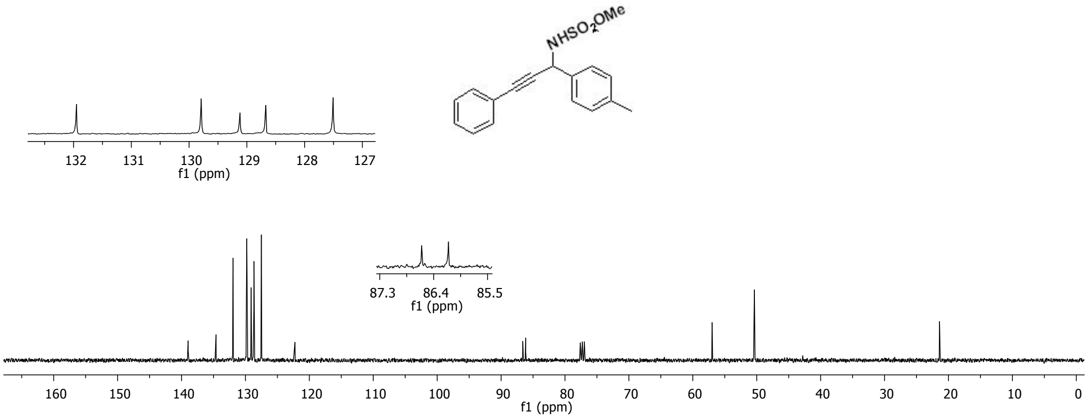

Compound 2e

^{1}H-NMR Spectrum of the (400 MHz, $CDCl_3$).

^{13}C-NMR Spectrum of the (100 MHz, $CDCl_3$).

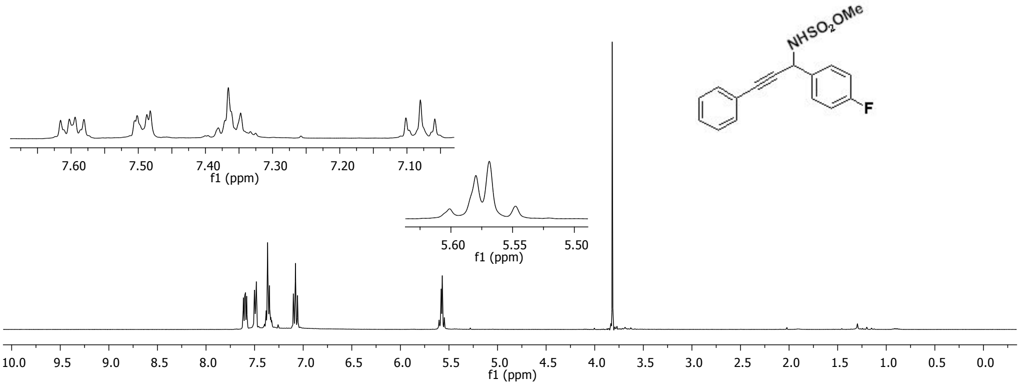

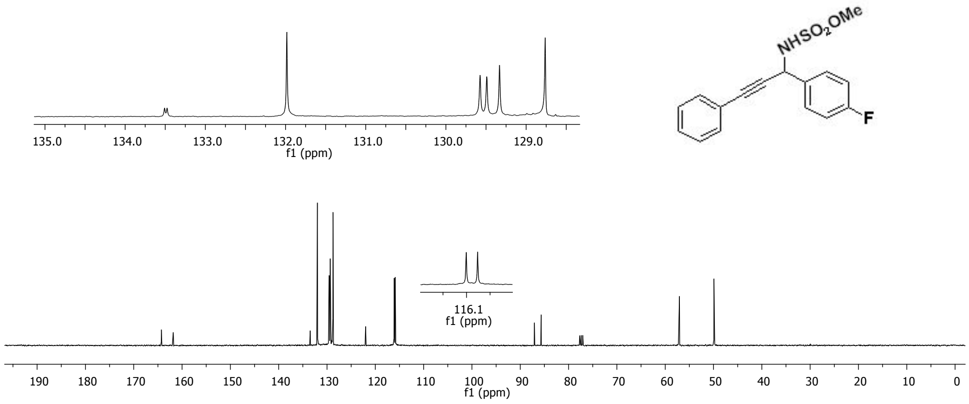

Compound 2f

^{1}H-NMR Spectrum of the (400 MHz, $CDCl_3$).

^{13}C-NMR Spectrum of the (100 MHz, $CDCl_3$).

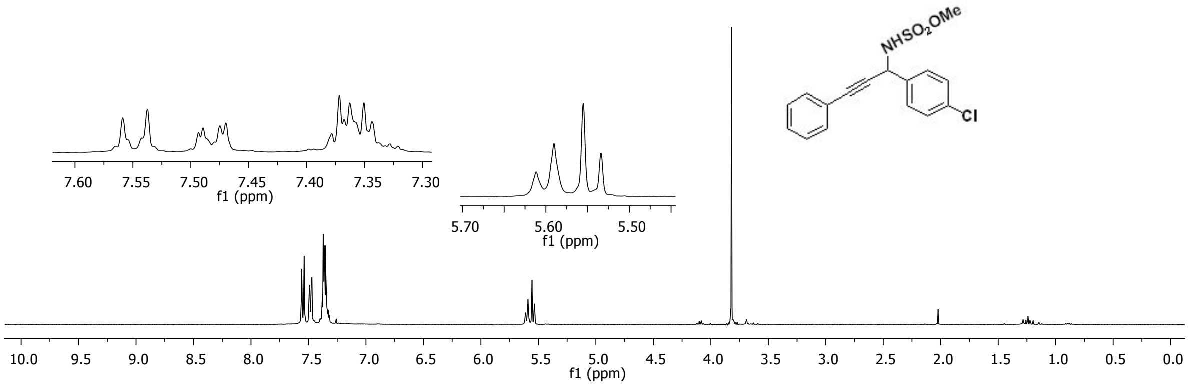

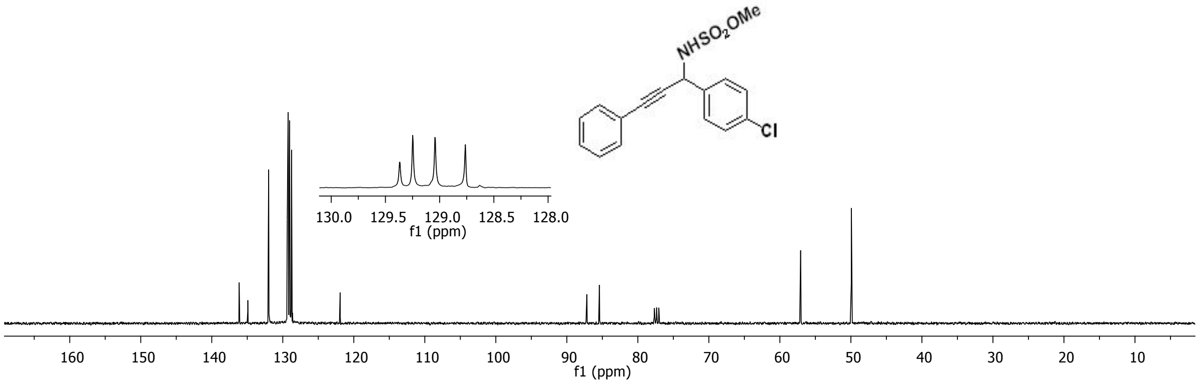

Compound 2g

^{1}H-NMR Spectrum of the (400 MHz, $CDCl_3$).

^{13}C-NMR Spectrum of the (100 MHz, $CDCl_3$).

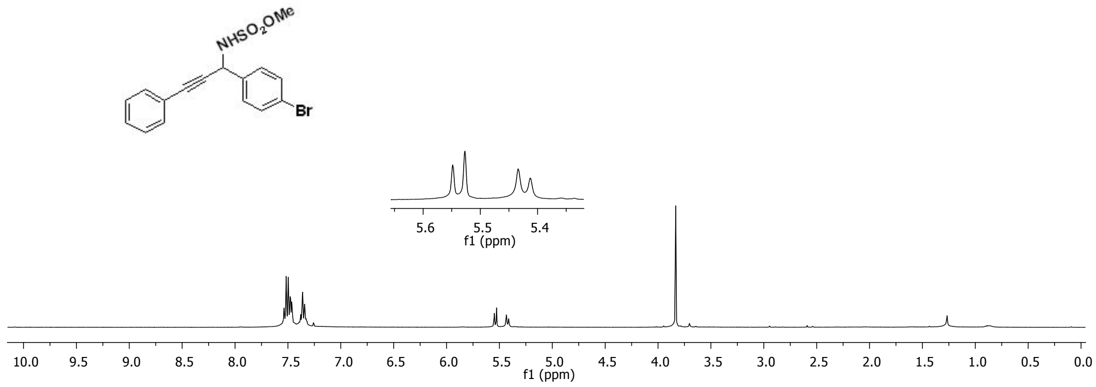

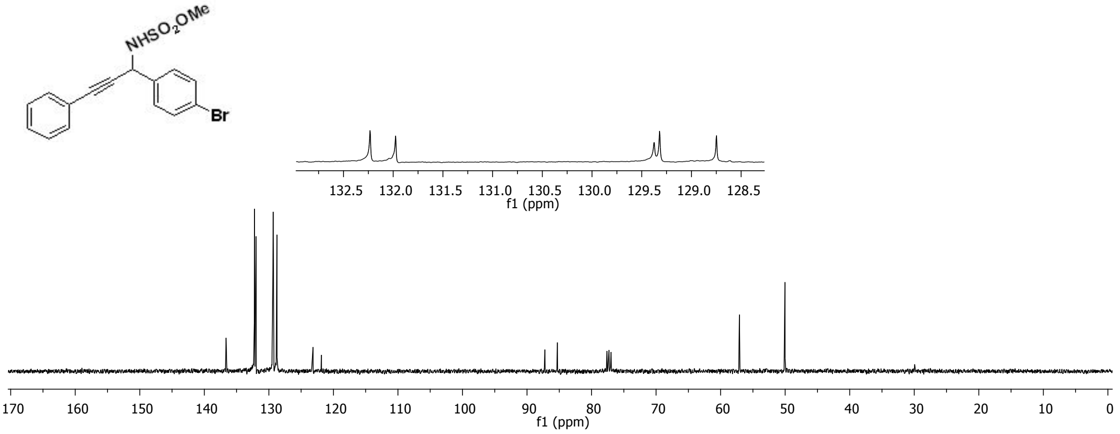

Compound 2h

^{1}H-NMR Spectrum of the (400 MHz, $CDCl_3$).

^{13}C-NMR Spectrum of the (100 MHz, $CDCl_3$).

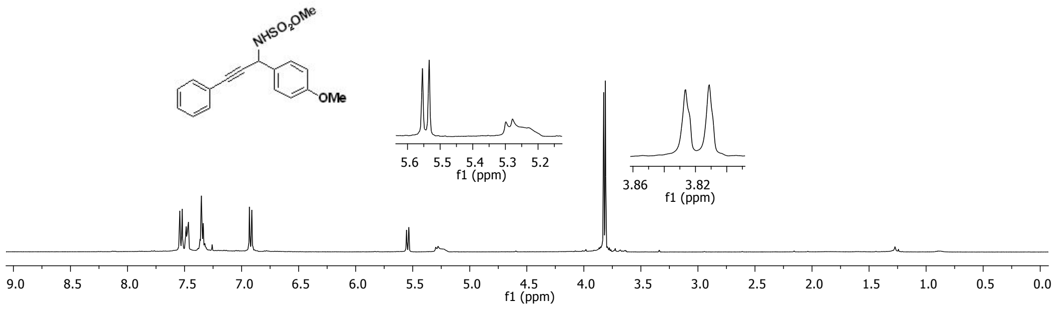

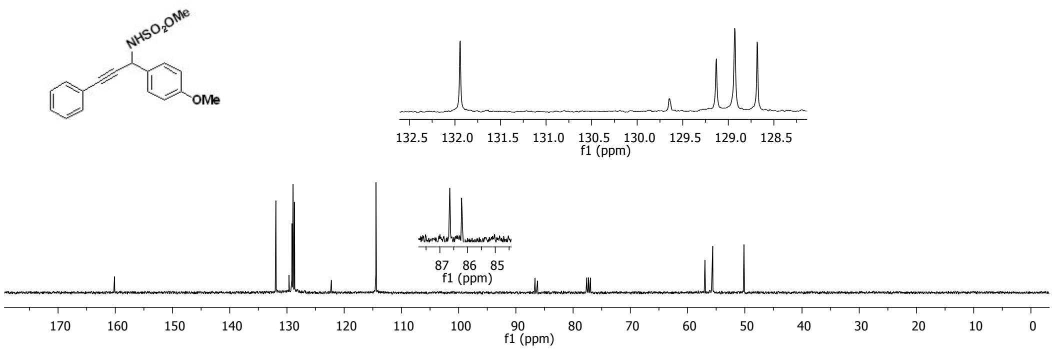

Compound 2i

^{1}H-NMR Spectrum of the (400 MHz, $CDCl_3$).

^{13}C-NMR Spectrum of the (100 MHz, $CDCl_3$).

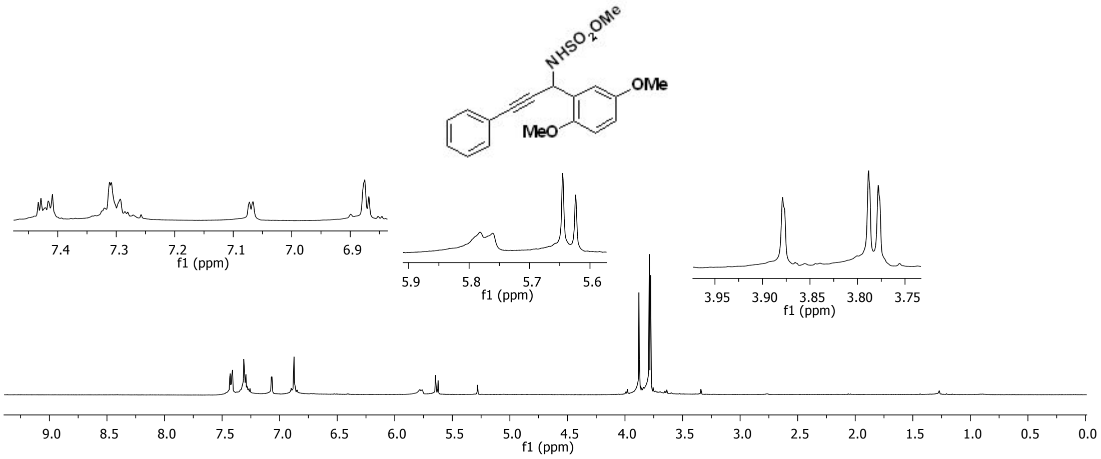

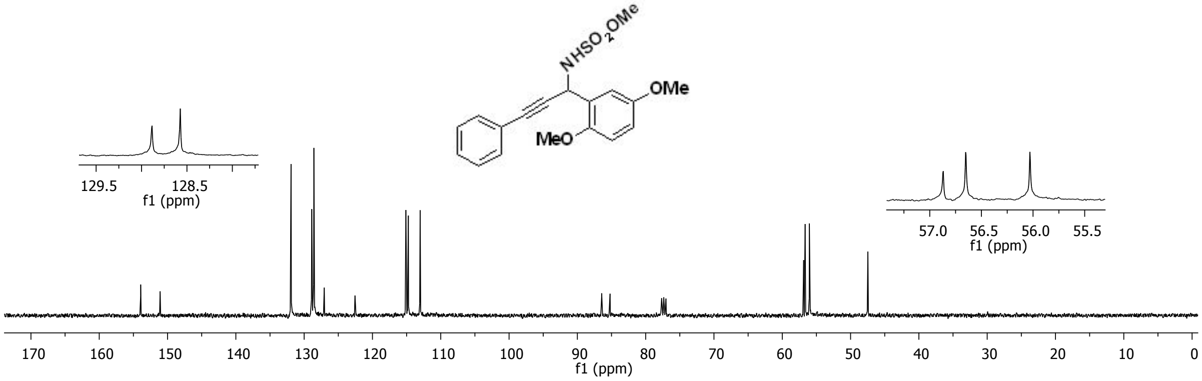

Compound 2j

^{1}H-NMR Spectrum of the (400 MHz, $CDCl_3$).

^{13}C-NMR Spectrum of the (100 MHz, $CDCl_3$).

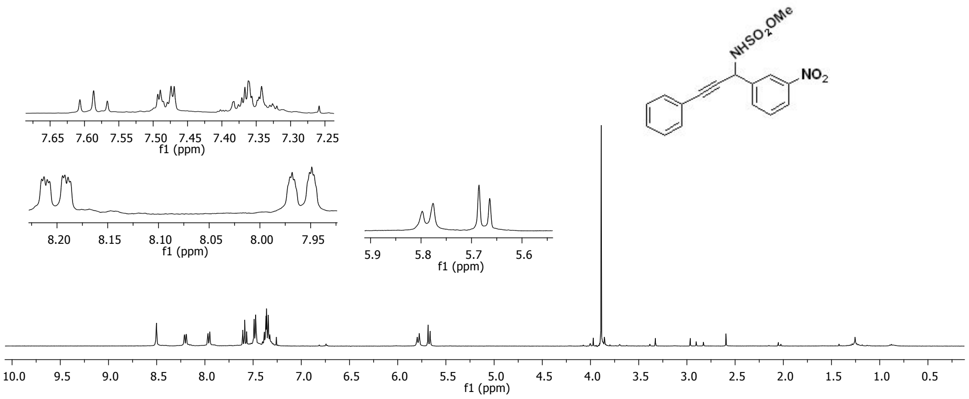

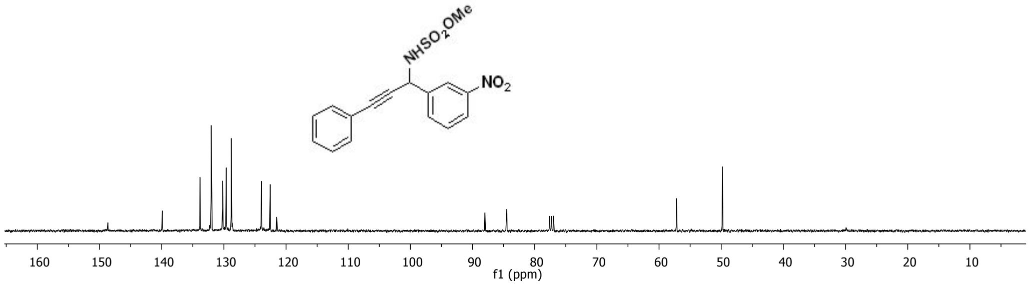

Compound 2k

^{1}H-NMR Spectrum of the (400 MHz, $CDCl_3$).

^{13}C-NMR Spectrum of the (100 MHz, $CDCl_3$).

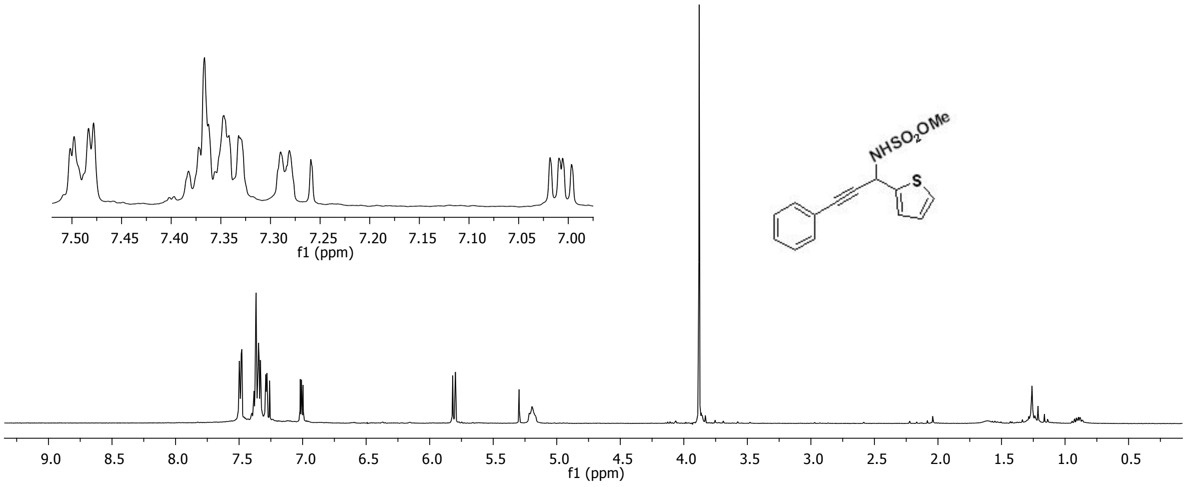

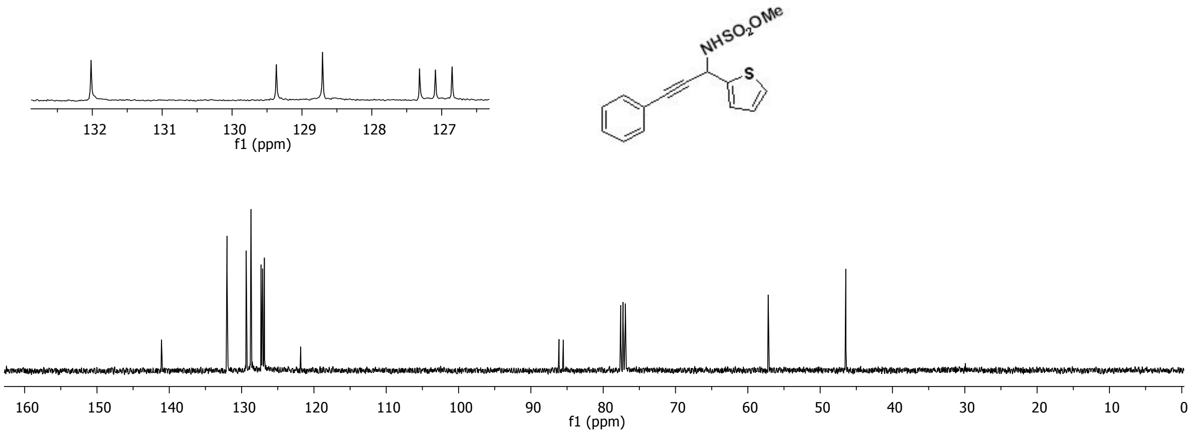

Compound 2l

^{1}H-NMR Spectrum of the (400 MHz, $CDCl_3$).

^{13}C-NMR Spectrum of the (100 MHz, $CDCl_3$).

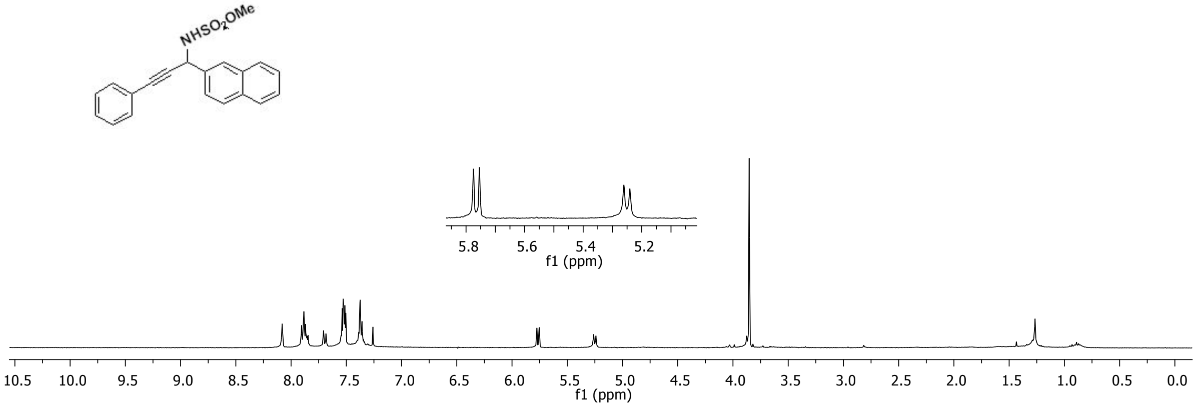

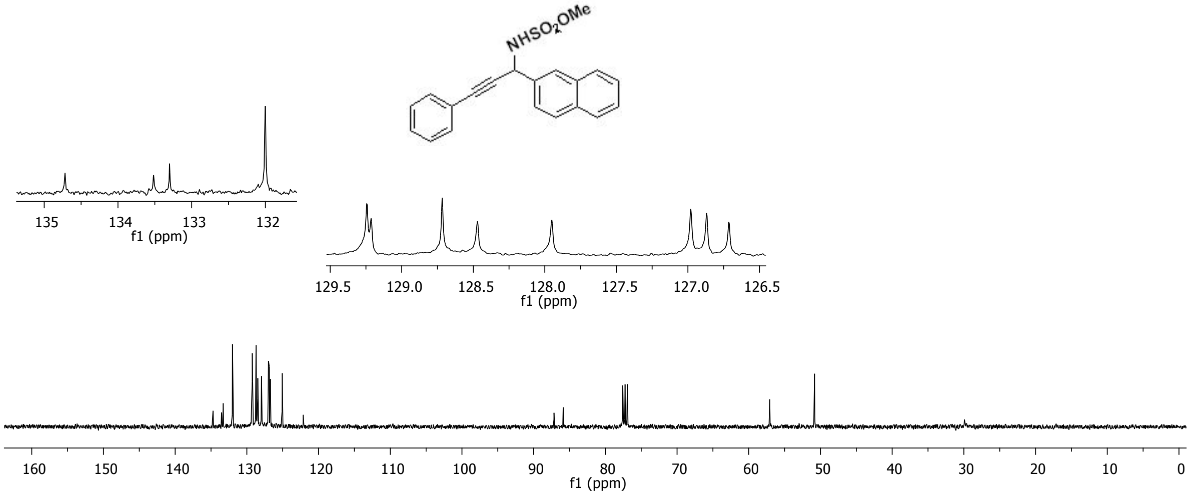

Compound 2m

^{1}H-NMR Spectrum of the (400 MHz, $CDCl_3$).

^{13}C-NMR Spectrum of the (100 MHz, $CDCl_3$).

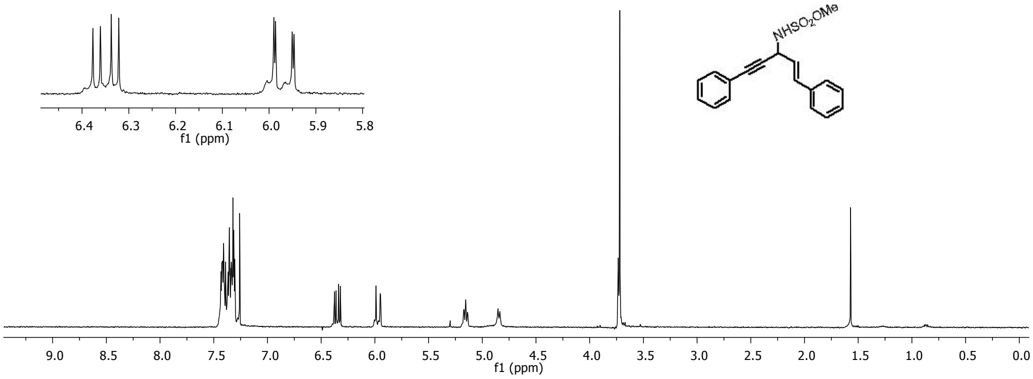

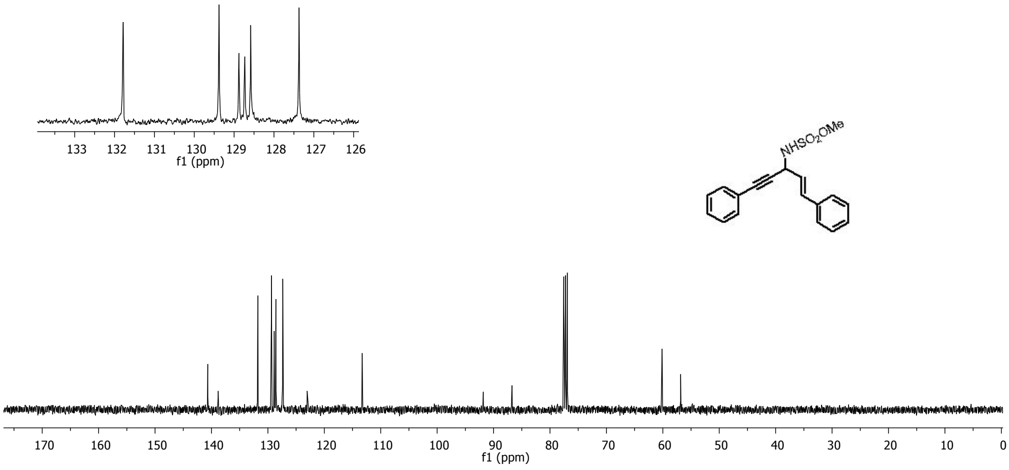

Compound 2n

^{1}H-NMR Spectrum of the (400 MHz, $CDCl_3$).

^{13}C-NMR Spectrum of the (100 MHz, $CDCl_3$).

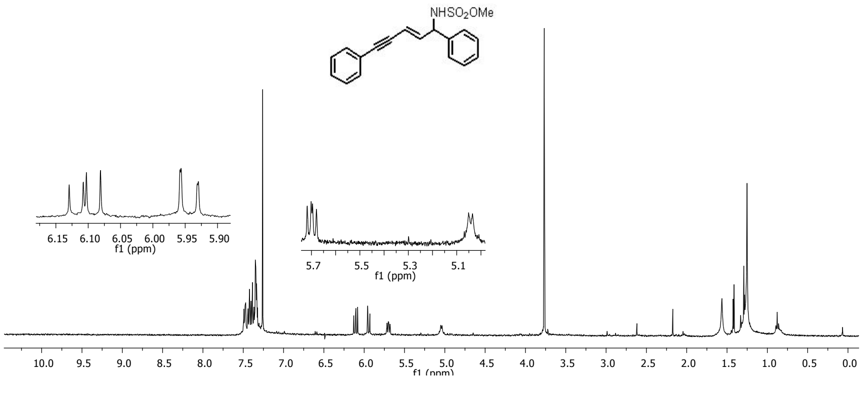

Compound 4a

^{1}H-NMR Spectrum of the (400 MHz, $CDCl_3$).

^{13}C-NMR Spectrum of the (100 MHz, $CDCl_3$).

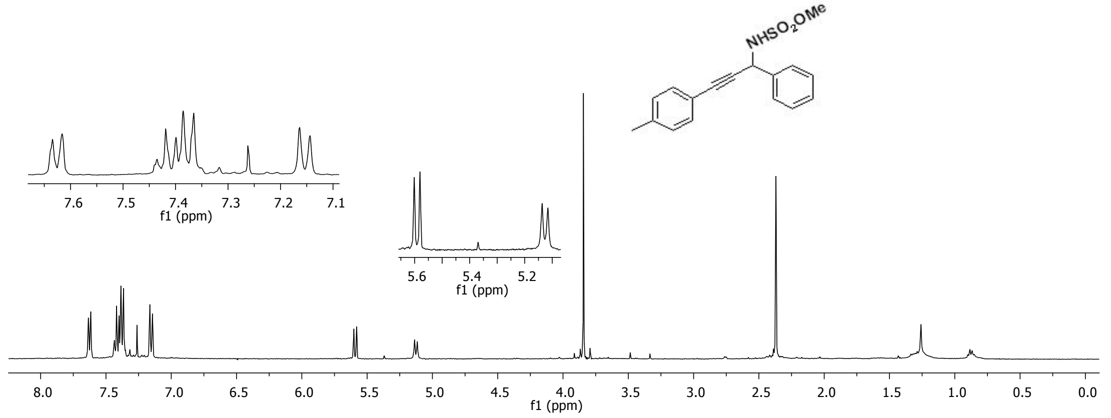

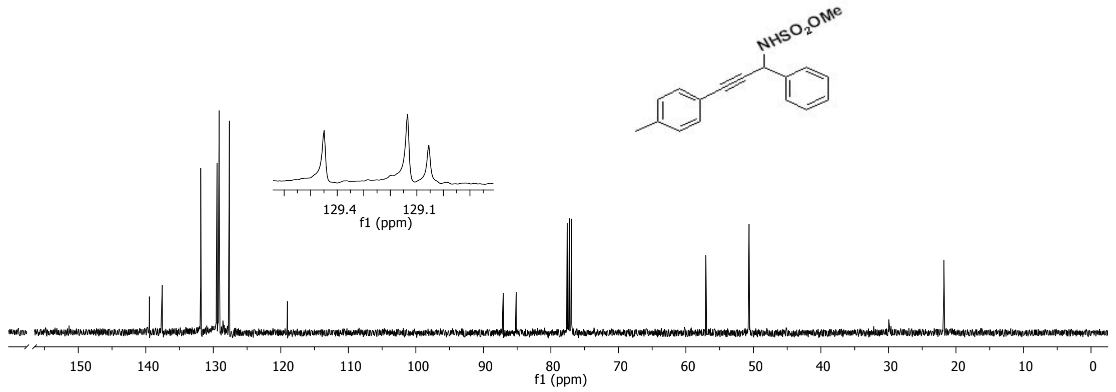

Compound 4b

^{1}H-NMR Spectrum of the (400 MHz, $CDCl_3$).

^{13}C-NMR Spectrum of the (100 MHz, $CDCl_3$).

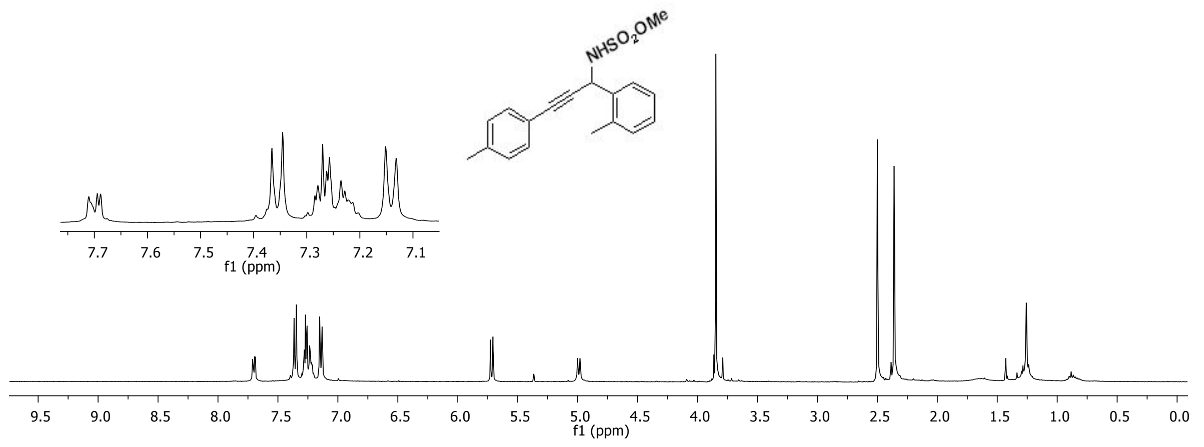

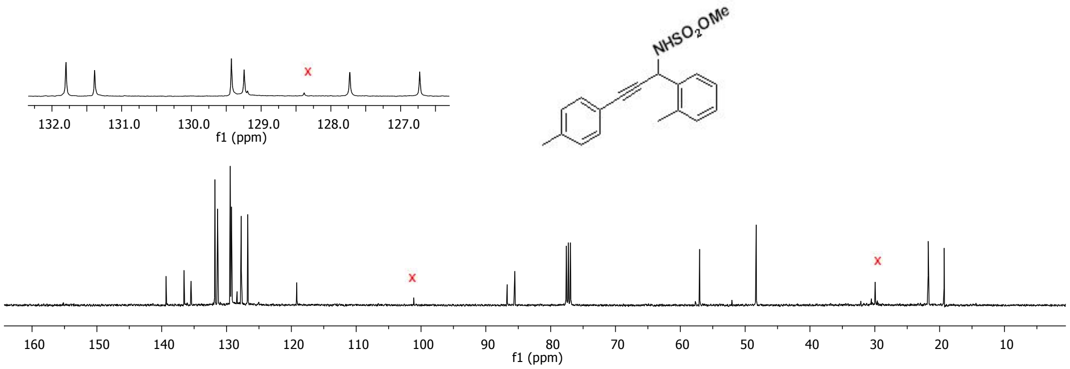

Compound 4c

^{1}H-NMR Spectrum of the (400 MHz, $CDCl_3$).

^{13}C-NMR Spectrum of the (100 MHz, $CDCl_3$).

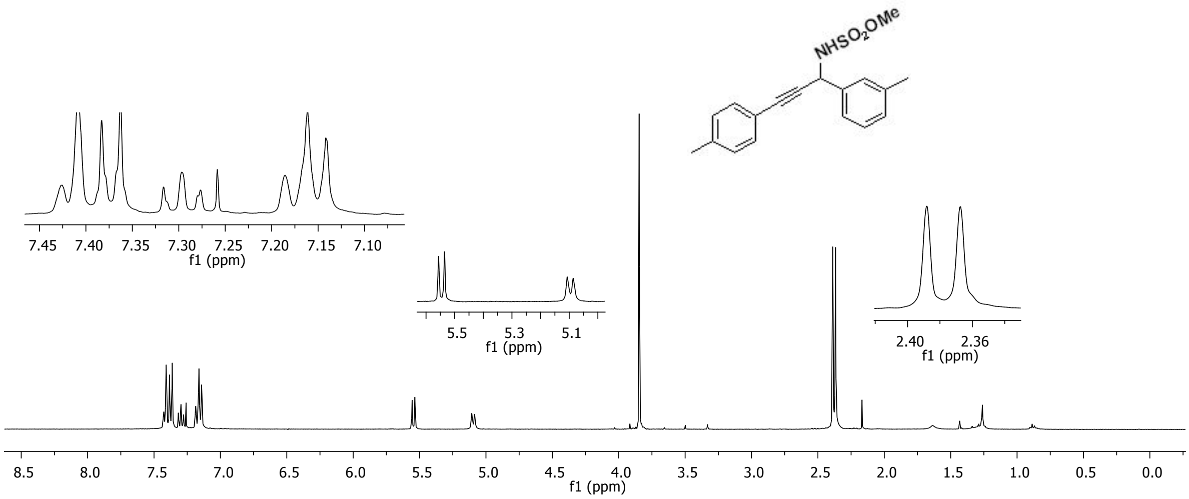

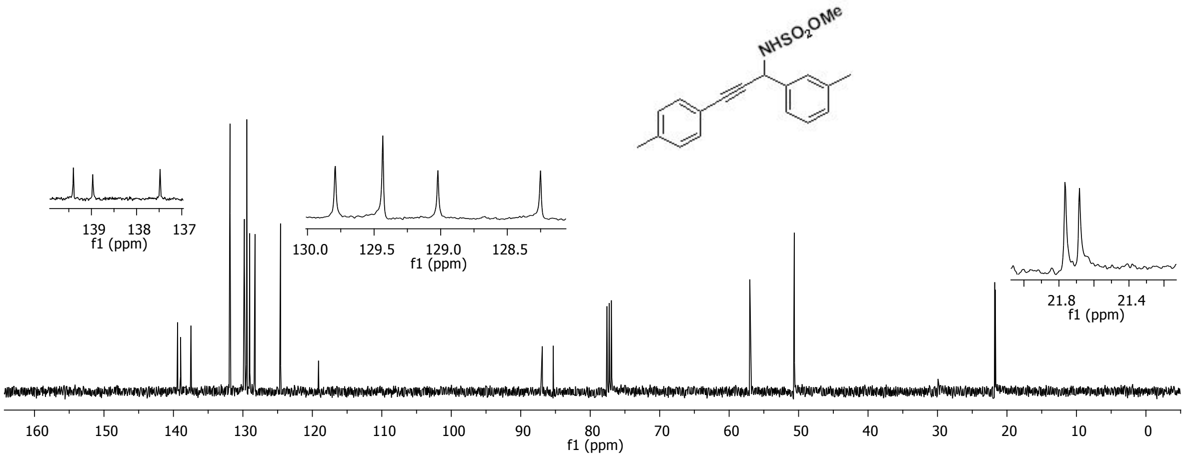

Compound 4d

^{1}H-NMR Spectrum of the (400 MHz, $CDCl_3$).

^{13}C-NMR Spectrum of the (100 MHz, $CDCl_3$).

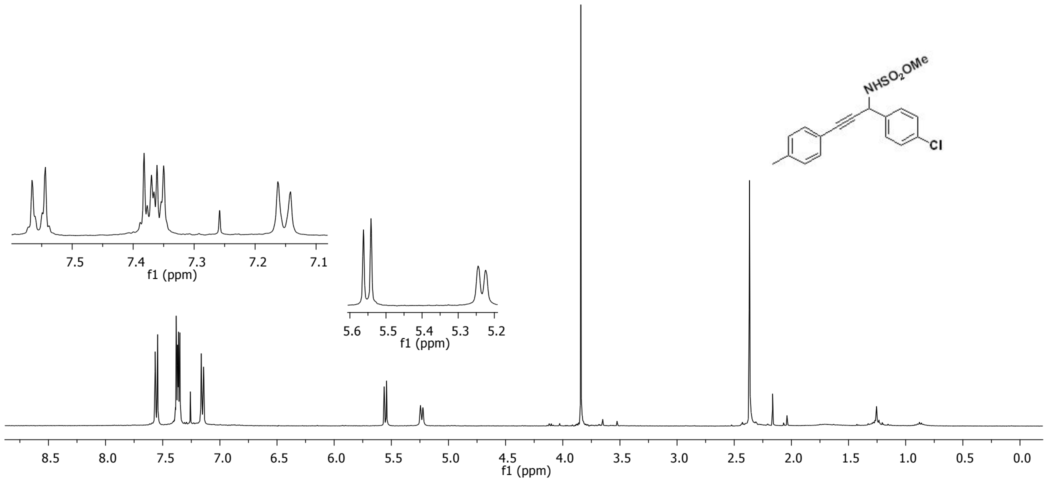

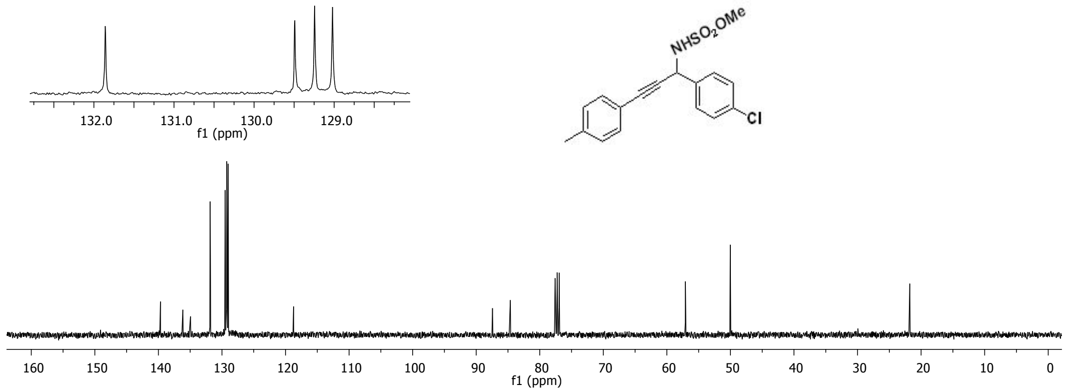

Compound 4e

^{1}H-NMR Spectrum of the (400 MHz, $CDCl_3$).

^{13}C-NMR Spectrum of the (100 MHz, $CDCl_3$).

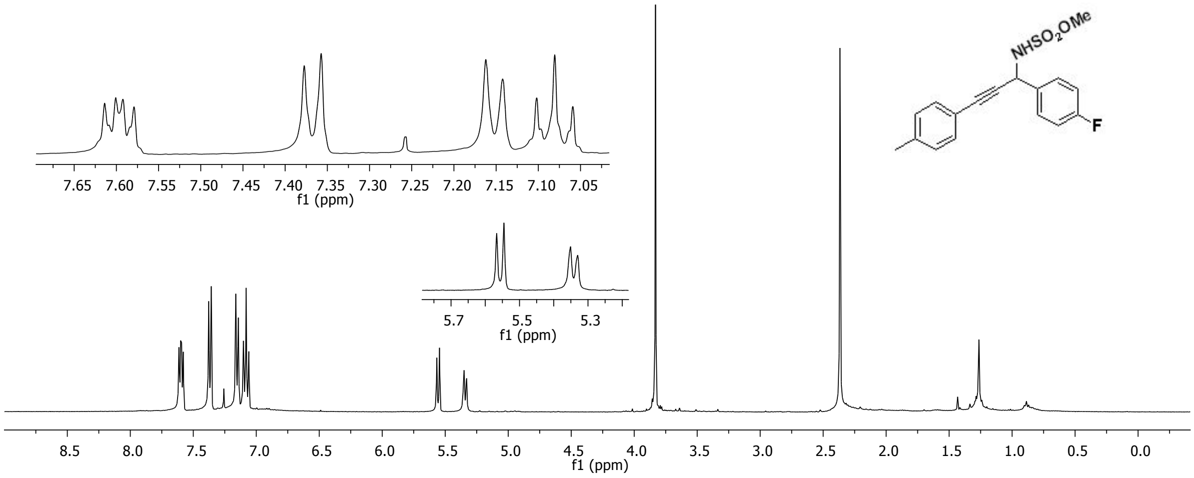

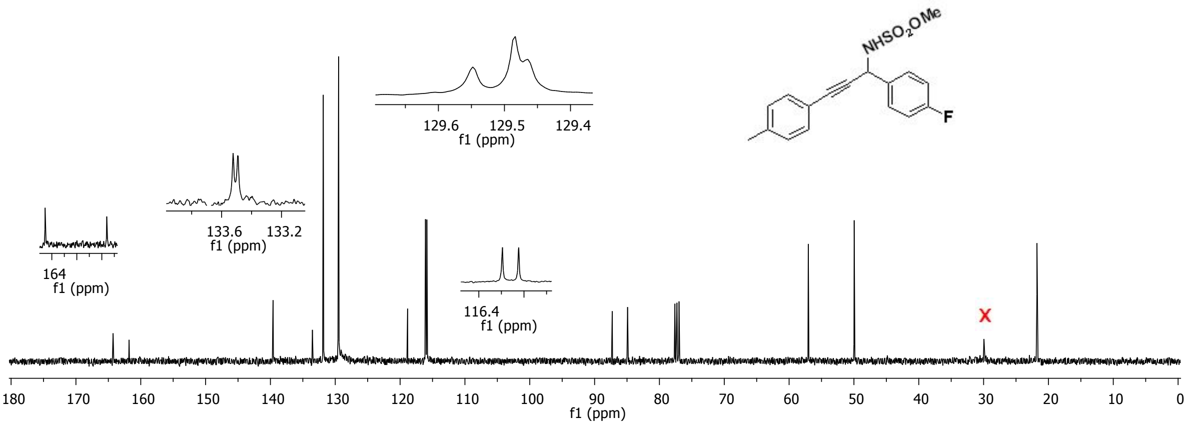

Compound 4f

^{1}H-NMR Spectrum of the (400 MHz, $CDCl_3$).

^{13}C-NMR Spectrum of the (100 MHz, $CDCl_3$).

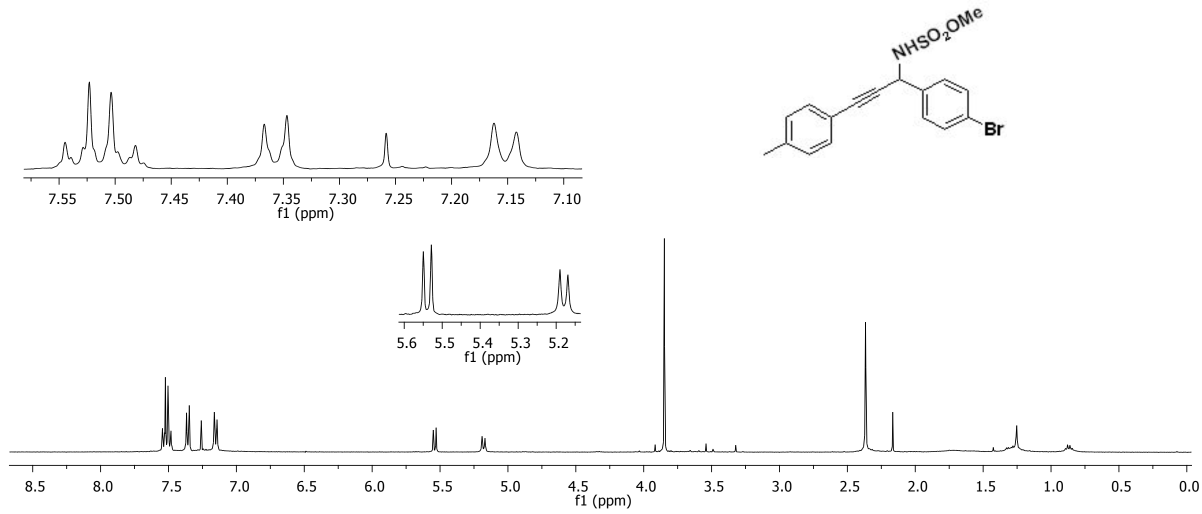

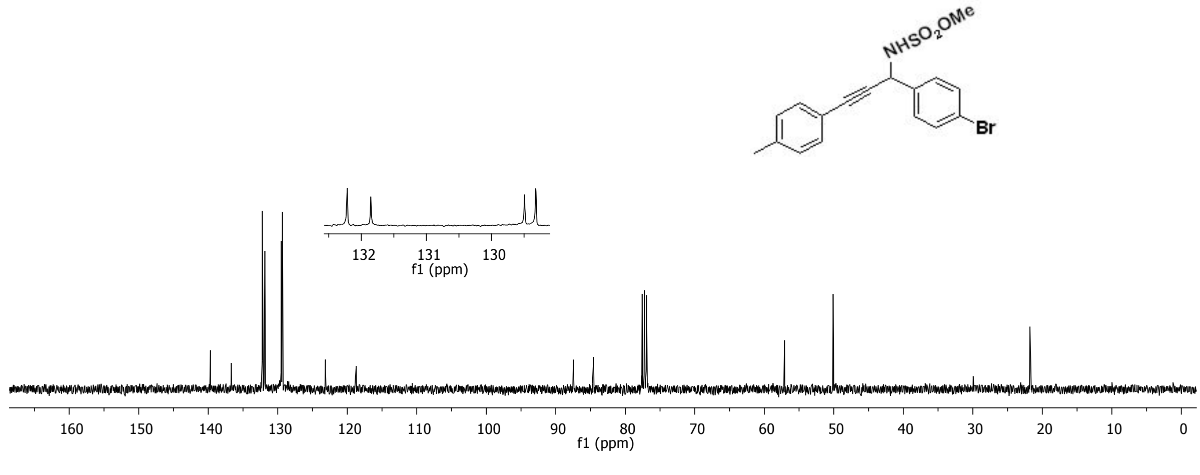

Compound 4g

^{1}H-NMR Spectrum of the (400 MHz, $CDCl_3$).

^{13}C-NMR Spectrum of the (100 MHz, $CDCl_3$).

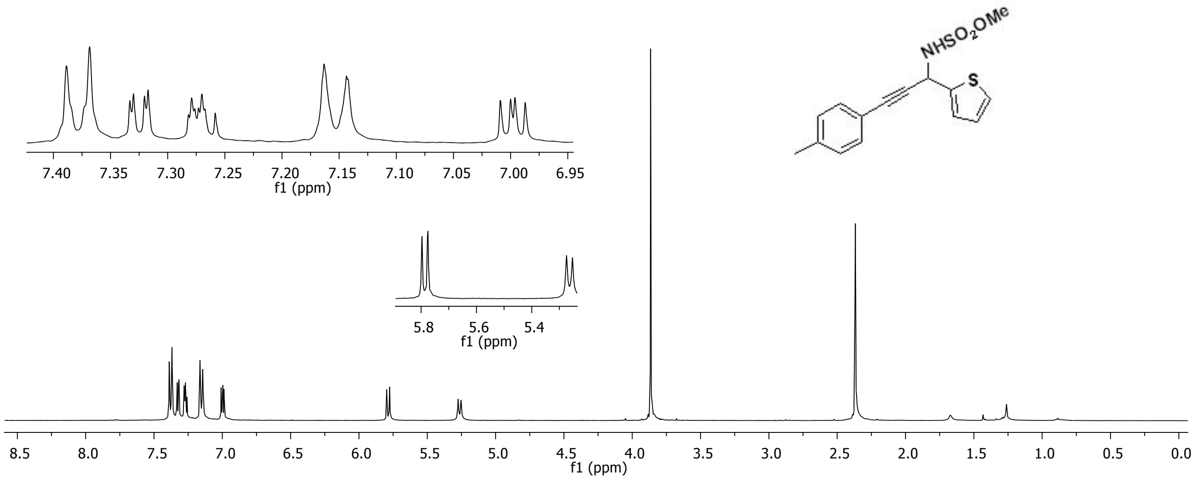

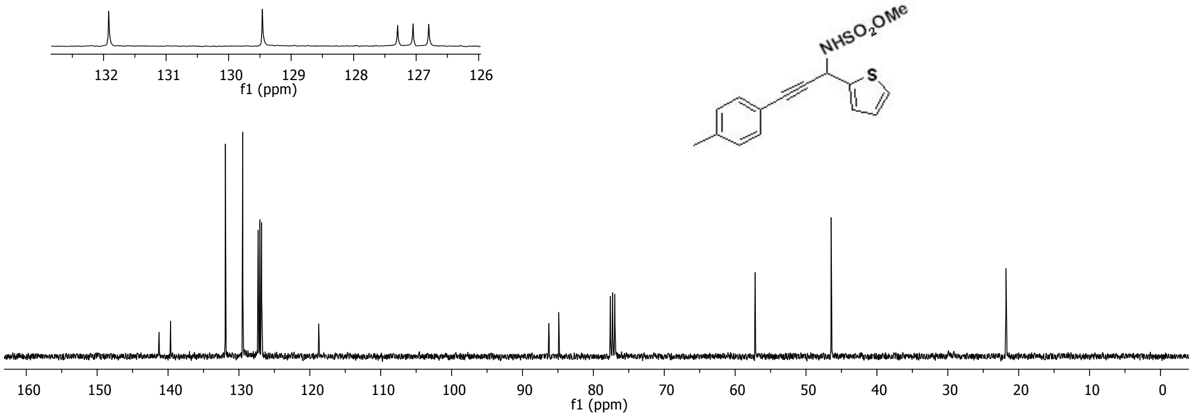

Supplement: Supplementary file 1 [file ao6c02129_si_001.pdf]
